# Complex c-di-GMP Signaling Networks Mediate Transition between Virulence Properties and Biofilm Formation in *Salmonella enterica* Serovar Typhimurium

**DOI:** 10.1371/journal.pone.0028351

**Published:** 2011-12-02

**Authors:** Irfan Ahmad, Agaristi Lamprokostopoulou, Soazig Le Guyon, Elena Streck, Melanie Barthel, Verena Peters, Wolf-Dieter Hardt, Ute Römling

**Affiliations:** 1 Department of Microbiology, Tumor and Cell Biology (MTC), Karolinska Institutet, Stockholm, Sweden; 2 Institute of Microbiology, ETH Zürich, Zürich, Switzerland; University of Birmingham, United Kingdom

## Abstract

Upon *Salmonella enterica* serovar Typhimurium infection of the gut, an early line of defense is the gastrointestinal epithelium which senses the pathogen and intrusion along the epithelial barrier is one of the first events towards disease. Recently, we showed that high intracellular amounts of the secondary messenger c-di-GMP in *S. typhimurium* inhibited invasion and abolished induction of a pro-inflammatory immune response in the colonic epithelial cell line HT-29 suggesting regulation of transition between biofilm formation and virulence by c-di-GMP in the intestine. Here we show that highly complex c-di-GMP signaling networks consisting of distinct groups of c-di-GMP synthesizing and degrading proteins modulate the virulence phenotypes invasion, IL-8 production and *in vivo* colonization in the streptomycin-treated mouse model implying a spatial and timely modulation of virulence properties in *S. typhimurium* by c-di-GMP signaling. Inhibition of the invasion and IL-8 induction phenotype by c-di-GMP (partially) requires the major biofilm activator CsgD and/or BcsA, the synthase for the extracellular matrix component cellulose. Inhibition of the invasion phenotype is associated with inhibition of secretion of the type three secretion system effector protein SipA, which requires c-di-GMP metabolizing proteins, but not their catalytic activity. Our findings show that c-di-GMP signaling is at least equally important in the regulation of *Salmonella*-host interaction as in the regulation of biofilm formation at ambient temperature.

## Introduction

*Salmonella enterica* serovar Typhimurium is a foodborne bacterial pathogen whose pathology in Man ranges from gastroenteritis to systemic disease [1]. This lifestyle of *S. typhimurium* requires adaptation and survival mechanisms inside and outside the host. Biofilm formation of *S. typhimurium* is established as a survival mechanism outside the host [2], [3], [4], but expression of biofilm components has also been observed in hosts [5], [6].

Outside hosts, the secondary messenger c-di-GMP reversely regulates sessility (biofilm formation) and motility in *S. typhimurium* [7]. In *S. typhimurium* and other bacteria intracellular concentrations of c-di-GMP are controlled through multiple GG(D/E)EF domain proteins acting as di-guanylate cyclases (DGCs) and EAL domain proteins acting as c-di-GMP specific phosphodiesterases (PDEs) [8], [9], [10].

At least eight of the 20 GG(D/E)EF/EAL domain proteins in *S. typhimurium* contribute directly or indirectly to the regulation of the rdar morphotype, a biofilm phenotype characterized by the expression of the extracellular matrix components cellulose and curli fimbriae [11], [12], [13]. The rdar morphotype is positively regulated by the transcriptional regulator CsgD that in turn, activates the curli biosynthesis operon *csgBAC* and *adrA*, encoding a di-guanylate cyclase which mediates cellulose biosynthesis [14]. Although the rdar morphotype is expressed outside the host at ambient temperature, we could recently show that high c-di-GMP levels inhibit the virulence properties invasion and induction of the pro-inflammatory cytokine IL-8 through expression of the biofilm regulator CsgD and/or the extracellular matrix components cellulose and the capsule at body temperature [15] suggesting that c-di-GMP regulates the transition between biofilm formation and virulence at the intestinal epithelial cell lining.

Upon infection of the gut, the gastrointestinal epithelium is an early line of defense and intrusion of *S. typhimurium* along the epithelial barrier is one of the first events towards disease [16]. One way of crossing the gastrointestinal epithelial cell barrier is invasion into the non-phagocytic epithelial cells [17]. Invasion of epithelial cells by *S. typhimurium* requires the type three secretion system encoded on the *Salmonella* pathogenicity island 1 (TTSS-1), which translocates more than 25 effector proteins, which cumulatively promote the uptake of the bacteria into host cells [18], [19], [20].

In addition, the ability of the epithelial cell lining to recognize the invading pathogen is crucial for triggering an appropriate immune response. Pathogen-associated molecular patterns (PAMPs) are recognized by Toll-like receptors (TLRs) on the surface of epithelial cells and stimulate the release of pro-inflammatory cytokines in order to promote subsequent immune responses [21]. A PAMP playing an important role as a danger signal is the protein flagellin, the monomeric subunit of flagella. Recognition of flagellin by TLR-5 is often studied in well-defined cell culture models with induction of IL-8 as a read out [22]. Models for human gastroenteritis, the most frequent disease manifestation upon *S. typhimurium* infection, are the streptomycin pretreated mice and the bovine colitis model [23], [24].

Although there is evidence of a contribution of c-di-GMP signaling to virulence of *S. typhimurium* [25], [26], regulation of virulence by c-di-GMP signaling in *S. typhimurium* has not been established. On the contrary, in a *S. enteriditis* strain with deletion of all GG(D/E)EF domain proteins, expression of the catalytically inactive di-guanylate cyclase STM4551 was sufficient to restore virulence in the typhoid fever mouse model, a systemic infection model [26]. Also the EAL-domain like protein STM1344, which prevents *Salmonella* induced macrophage killing and mediates resistance to oxidative stress [25], neither metabolizes nor binds c-di-GMP [13]. In contrast, c-di-GMP signaling has been shown to modulate virulence in the enteric pathogen *Vibrio cholerae*, the causative agent of cholera [27]. Mostly based on studies in *V. cholerae* and *P. aeruginosa* the common view aroused that c-di-GMP is promoting chronic infections, while inhibiting acute infections [28], [29].

In this study, we identified among the 20 GG(D/E)EF and EAL domain proteins distinct groups whose members additively and redundantly affect the virulence phenotypes invasion of epithelial cells and induction of IL-8 production in epithelial cells mainly through c-di-GMP signalling, but also independently of c-di-GMP. Inhibition of invasion is associated with lack of secretion of the TTSS-1 effector protein SipA and partially requires the biofilm regulator CsgD and the cellulose synthase BcsA, while inhibition of IL-8 induction is mediated through CsgD. In addition, three GG(D/E)EF/EAL domain proteins showed a phenotype in gut colonization in the streptomycin-treated mouse model. In summary, this work shows the delicate regulation of virulence phenotypes by the c-di-GMP signaling network in *S. typhimurium*.

## Results

### Identification of a subset of GG(D/E)EF/EAL domain proteins leading to alteration of invasion

**Figure 1:**
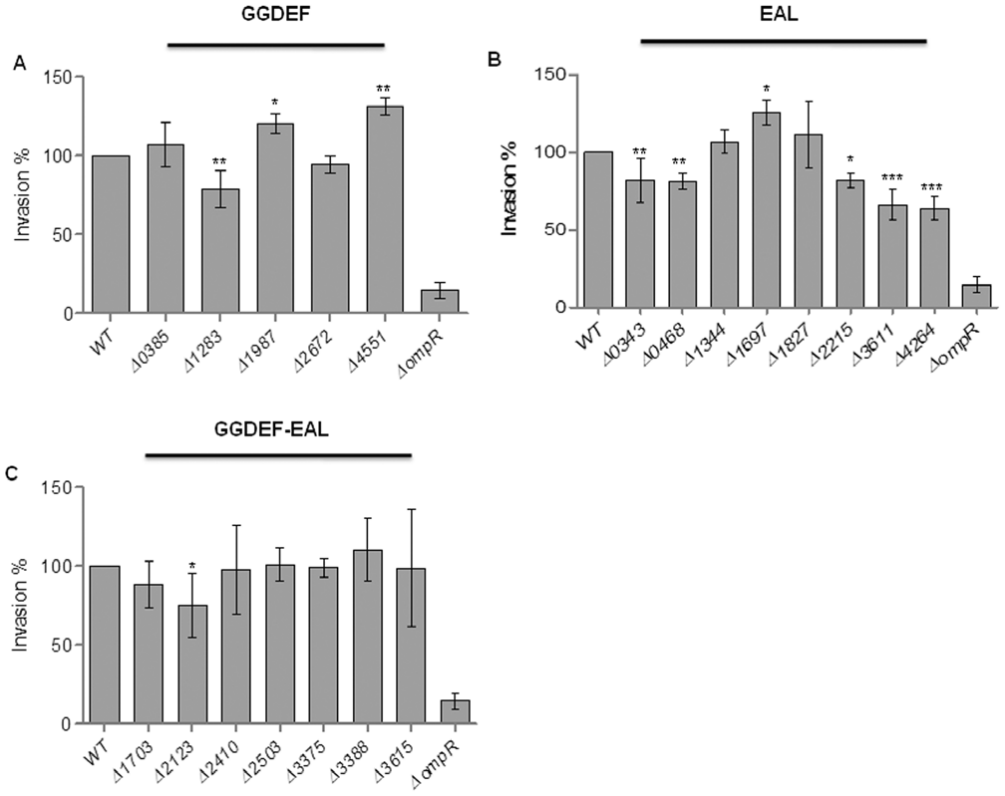
Individual GG(D/E)EF/EAL domain proteins affect *S. typhimurium* UMR1 invasion into HT-29 gastrointestinal epithelial cells. Invasion of (A) GG(D/E)EF-domain protein mutants, of (B) EAL-domain protein mutants and (C) GG(D/E)EF-EAL-domain protein mutants relative to the wild type *S. typhimurium* UMR1. Strains were grown under invasion inducing conditions (standing culture, LB+0.3M NaCl) until O.D.600 0.6. HT-29 cells were infected for 1 h with 10^7^ CFU and subsequently incubated for an additional hour with 100 µg/ml gentamicin to kill extracellular bacteria. Δ*ompR*, negative control for invasion. Invasion % is defined as (invasion value of mutant/invasion value of wild type)*100, whereby for each strain invasion is calculated as (CFU recovered inside epithelial cells/CFU at time of inoculation). Bars show mean ± standard deviation from at least five independent biological experiments performed in two technical replicates. Statistical significance is indicated by *P<0.05; **P<0.01; ***P<0.001 as compared with wild type *S. typhimurium* UMR1 (WT).

Our previous results showed that c-di-GMP signaling inhibits invasion as saturation of the cell with c-di-GMP through overexpression of the diguanylate cyclase AdrA leads to inhibition of the invasion phenotype of *S. typhimurium* into the colonic epithelial cell line HT-29 [15]. To identify the relevant chromosomally encoded c-di-GMP metabolizing proteins, we investigated the impact of mutants of the 20 GG(D/E)EF/EAL domain proteins of *S. typhimurium*, most of them putative or demonstrated c-di-GMP metabolizing proteins (Table 1; Fig. S1; [11], [12]), on the invasion phenotype. Surprisingly, 10 of 20 mutants showed a significantly altered invasion phenotype (Fig. 1).

**Table 1:** Function of GG(D/E)EF/EAL domain proteins in *Salmonella typhimurium*.

GGDEF domain proteins	Function	EAL domain proteins	Function	GGDEF-EAL domain proteins	Function
STM0385 (AdrA)	DGC [7]	STM0343	predicted PDE	STM1703	PDE [12], [66]
STM1283	predicted DGC	STM0468	predicted PDE	STM2123	DGC [11]
STM1987	DGC [7]	STM1344	No PDE, no DGC, noc-di-GMP binding [13]	STM2410	predicted PDE
STM2672	predicted DGC	STM1697	-	STM2503	predicted PDE
STM4551	DGC [26]	STM1827	PDE [12]	STM3375	No PDE, no DGC, noc-di-GMP binding [58]
		STM2215	predicted PDE	STM3388	DGC [11]
		STM3611	PDE [7]	STM3615	predicted PDE
		STM4264	PDE [12]		

First, the mutants with deletion of a GG(D/E)EF domain protein were screened for the ability to invade HT-29 cells. As GG(D/E)EF domain proteins are proven or predicted diguanylate cyclases (Table 1), we expected deletion of a GG(D/E)EF domain gene to lead to a reduced overall or local c-di-GMP production and consequently to a higher invasion rate as the phenotype output. Of 5 mutants, the STM1283, STM1987 and STM4551 mutants showed a significant alteration of invasion (Fig. 1A). The STM4551 and the STM1987 mutant showed an invasion rate significantly higher than the wild type (131±4% and 120±5% of wild type *S. typhimurium* UMR1, respectively). This finding is consistent with the previously demonstrated di-guanylate cyclase activity of the two proteins [7], [26]. At the same time though, the deletion of STM1283 showed a 21±8% reduction of the invasion rate compared with wild type *S. typhimurium* UMR1, thus displaying an unsuspected or unconventional phenotype. Other GGDEF-domain protein deletions had no significant effect on invasion.

Next, we screened the deletion mutants of EAL-domain proteins for their invasion phenotype. As most of the EAL domain proteins have predicted or demonstrated c-di-GMP specific phosphodiesterase activity (Table 1; Fig. S1; [12]), we expected inhibition of invasion as the mutant phenotype. Six of eight mutants, the STM0343, STM0468, STM1697, STM2215, STM3611 and STM4264 mutants, demonstrated a statistically significant alteration of the invasion phenotype (Fig. 1B). The STM3611 and STM4264 mutants showed the lowest invasion rate, reduced to 66±10% and 64±8% of wild type *S. typhimurium* UMR1 levels, respectively. The invasion rate of the STM0343, STM0468 and STM2215 mutants was significantly reduced by 18±8%, 19±4% and 18±3% respectively, compared to wild type UMR1. Interestingly, the STM1697 mutant showed a 26±6% higher invasion rate thus displaying an unconventional phenotype.

Screen of the 7 GG(D/E)EF-EAL protein mutants demonstrated a significantly reduced invasion of the STM2123 mutant (33±10% reduction compared with the wild type *S. typhimurium* UMR1) (Fig. 1C). Previously, we demonstrated that STM2123 activated expression of the biofilm regulator CsgD and showed di-guanylate cyclase activity *in vivo* [11]. In addition, its EAL domain does not show the signature of an active c-di-GMP specific phosphodiesterase (Fig. S1B). Thus, the STM2123 mutant has an unconventional invasion phenotype which cannot be caused by polar effects as *STM2123* is a stand-alone gene.

### A regulatory role of the c-di-GMP signaling network in the invasion phenotype

**Figure 2:**
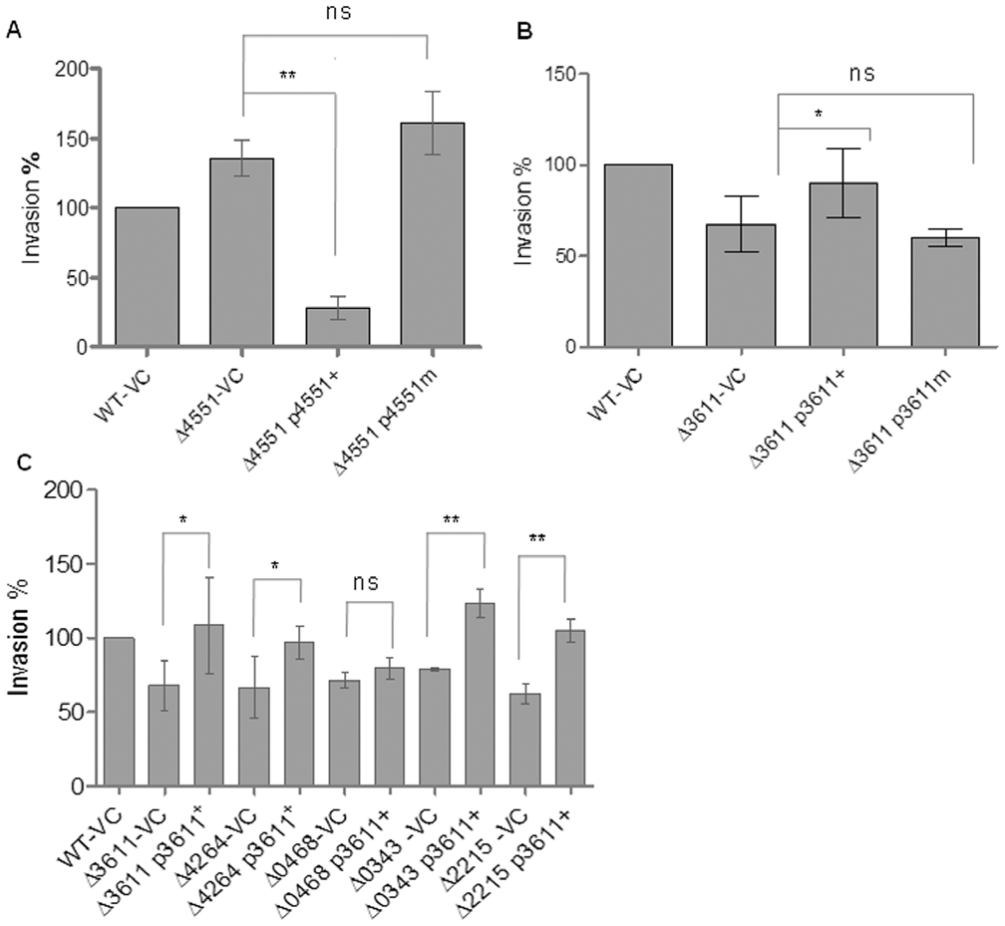
Complementation of the invasion phenotype of GG(D/E)EF/EAL protein mutants. (A) Complementation of the STM4551 mutant with STM4551 and STM4551_E267A_ demonstrates requirement of the di-guanylate cyclase activity of STM4551 for complementation of the invasion phenotype. The enhanced invasion rate exhibited by the STM4551 mutant was significantly reduced by complementation with wild type STM4551 (p4551+), but not with the non-functional mutant STM4551_E267A_ (p4551m) with the GGEEF motif altered to GGAEF. WT = wild type *S. typhimurium* UMR1; VC = vector control pBAD30. (B) Complementation of the STM3611 mutant with STM3611 and STM3611_K179A_ demonstrates requirement of the c-di-GMP phosphodiesterase activity of STM3611 for complementation of the invasion phenotype. The reduced invasion rate of the STM3611 mutant was significantly enhanced by complementation with wild type STM3611 (p3611+), but not with the non-functional mutant STM3611_K179A_ (p3611m). WT = wild type *S. typhimurium* UMR1; VC = vector control pBAD30. (C) The reduced invasion rate of EAL domain protein mutants of *S. typhimurium* was restored to wild type level by complementation with the EAL-domain only phosphodiesterase STM3611 in plasmid pLAFR3 (p3611+). No complementation was achieved in the STM0468 mutant. WT = wild type *S. typhimurium* UMR1. VC = vector control pLAFR3. Experimental conditions as in Figure 1. Bars show mean ± standard deviation from at least three independent biological experiments performed in two technical replicates. Statistical significance is indicated by **P<0.01; ***P<0.001 as compared with the corresponding mutant vector control.

To unambiguously couple the phenotype of a GG(D/E)EF/EAL domain protein mutant with the deletion, we performed complementation and mutation studies. We complemented the hyper-invasion phenotype of the STM4551 deletion mutant with STM4551 cloned in the vector pBAD30 under the control of the arabinose-inducible promoter. Expression of STM4551 abolished the hyper-invasion phenotype of the STM4551 mutant and lead to an invasion rate as low as 30±5% of the wild type (Fig. 2A). Consistent with this phenotype, we detected 1.75-fold higher c-di-GMP levels compared to the wild type *S. typhimurium* UMR1, when STM4551 was over-expressed under invasion conditions (Fig. S2). To demonstrate that the di-guanylate cyclase activity of STM4551 is required for repression of the invasion phenotype, the GGE_267_EF motif of STM4551 was altered to GGAEF to abolish the catalytic activity. E_267_ is the predicted catalytic base, but any amino acid mutation in the GG(D/E)EF motif abolishes the catalytic activity [30]. When we overexpressed STM4551_E267A_ under invasion conditions, we detected no rise in c-di-GMP levels compared to the wild type *S. typhimurium* UMR1 (Fig. S2) which demonstrated highly reduced or absent catalytic activity. Expression of STM4551_E267A_ in the STM4551 mutant did not repress the hyper-invasion phenotype (Fig. 2A) which demonstrated that STM4551 inhibits invasion through its di-guanylate cyclase activity.

We were not able to clone STM1987 without mutations. Expression of the di-guanylate cyclase STM4551, however, but not the STM4551_E267A_ mutant abolished the hyper-invasion phenotype of the STM1987 mutant (data not shown) showing the requirement of the di-guanylate cyclase activity for phenotype expression in the STM1987 mutant. In conclusion, the secondary messenger c-di-GMP is required to inhibit invasion. The STM1283 mutant with the unconventional phenotype will be analyzed in detail elsewhere.

In order to determine whether the reduction of invasion was due to the phosphodiesterase activity of the deleted EAL domain proteins, we complemented the STM3611 deletion mutant. Expression of the c-di-GMP specific phosphodiesterase STM3611 [7] from plasmid pBAD30 restored the invasion of the STM3611 mutant to wild type levels (Fig. 2B). We created the catalytically inactive mutant STM3611_K179A_. Exchange of the conserved lysine in the KIDRTF submotif was recently shown to abolish the catalytic activity of the RocR phosphodiesterase >10^5^-fold (Fig. S1; [31], [32]). Expression of STM3611_K179A_ maintained an invasion rate as low as the STM3611 mutant (Fig. 2B) showing that degradation of c-di-GMP is required for restoration of invasion in the STM3611 mutant.

We were not able to clone STM4264 without mutations [12]. In order to demonstrate the requirement of c-di-GMP degradation for complementation of the other EAL mutants, we expressed the EAL-only protein STM3611 and its catalytically inactive mutant STM3611_K179A_ in all EAL domain protein mutants with a conventional phenotype (reduced invasion upon deletion of the phosphodiesterase gene). The STM1697 mutant with an unconventional phenotype will be analyzed in detail elsewhere. The EAL domain protein STM3611 was chosen, as it is an EAL-only protein with its EAL domain most distantly related to all other EAL domains from *S. typhimurium* [14]. Expression of STM3611 in the STM0343, STM2215, and STM4264 mutants restored invasion to wild type levels (Fig. 2C), whereas by expression of STM3611_K179A_, the mutants maintained their low invasion rates (Fig. S3). Consequently, we conclude that the phosphodiesterase activity of the EAL domain is required for phenotype expression. In summary, these results clearly showed that c-di-GMP signaling is involved in the regulation of the invasion phenotype in *S. typhimurium*. Invasion was not recovered in the STM0468 mutant upon expression of STM3611 suggesting that STM0468 does not affect invasion through the predicted c-di-GMP specific phosphodiesterase activity.

### C-di-GMP metabolizing proteins affect secretion of the TTSS-1 effector protein SipA

The TTSS-1 is essential for invasion of epithelial cells [18], [19]. We have previously shown that overexpression of the di-guanylate cyclase AdrA inhibits secretion of the TTSS-1 effector protein SopE2 expressed from a plasmid under an IPTG-inducible promoter [15]. Here, we investigate the effect of individual mutants in c-di-GMP metabolizing proteins on secretion of SipA (*Salmonella* invasion protein), another TTSS-1 effector protein. SipA is injected into host cells promoting actin polymerisation, but can also be secreted into the intestinal lumen [33], [34]. We used strains with a SipA–beta-lactamase fusion protein expressed from its native chromosomal location [35] to cover potential transcriptional and post-transcriptional regulation by c-di-GMP.

**Figure 3:**
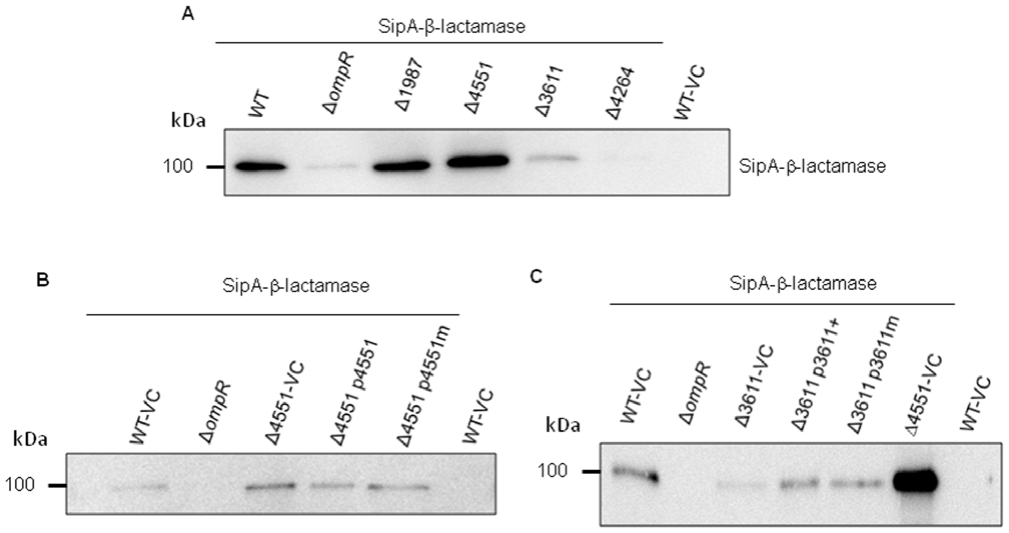
GG(D/E)EF/EAL domain proteins affect secretion of the SipA effector protein. (**A**) Mutants of the diguanylate cyclases STM1987 and STM4551 show enhanced secretion of a SipA-β-lactamase fusion protein, while secretion was diminished in mutants of the phosphodiesterases STM3611 and STM4264. (B) Enhanced secretion of the SipA-β-lactamase fusion protein in the STM4551 mutant is restored by expression of STM4551 from plasmid pBAD30 and by expression of the catalytically inactive STM4551_E267A_ mutant. (C) Diminished secretion of the SipA-β-lactamase fusion protein in the STM3611 mutant is restored by expression of STM3611 from plasmid pBAD30 and by expression of the catalytically inactive STM3611_K179A_ mutant. Positive control = Δ*4551*-VC. SipA-β-lactamase indicates chromosomal fusion in respective strains. Detection of the SipA-β-lactamase fusion protein by western blot analysis using an anti-β-lactamase antibody. Strain *S. typhimurium* UMR1 with pBAD30 expressing β-lactamase in the periplasm (WT-VC) served as β-lactamase secretion control. WT = wild type *S. typhimurium* UMR1; Δ*ompR*, negative control; VC = vector control pBAD30.

First, we investigated SipA secretion in the STM4551 and STM1987 mutants, as these two di-guanylate cyclases negatively affect the invasion phenotype. In comparison to the wild type, secretion of SipA was increased in the STM1987 and STM4551 mutants (Fig. 3A), but unaltered in a GG(D/E)EF/EAL mutant, which did not affect invasion (data not shown). Plasmid-expressed STM4551 and the catalytically inactive STM4551_E267A_ mutant complemented the hyper-secretion phenotype of the STM4551 and STM1987 mutants (Fig. 3B and data not shown). These results suggest that the GGDEF domain protein STM4551, but not its di-guanylate cyclase activity is required for inhibition of secretion.

Secretion of SipA was also investigated in the EAL domain proteins mutants STM3611 and STM 4264 as those EAL domain protein mutants showed the strongest inhibition of invasion. In addition, STM3611 and STM4264 do not show redundant function (see next paragraph). SipA secretion was reduced in the EAL domain protein mutants (Fig. 3A). Plasmid-expressed STM3611 and the catalytically inactive STM3611_K179A_ mutant complemented the hypo-secretion phenotype of the STM3611 mutant (Fig. 3C). These results suggest that the EAL domain protein STM3611, but not its c-di-GMP phosphodiesterase activity is required for stimulation of secretion.

Taken together, these data are consistent with an inhibitory role of the GGDEF domain and a stimulating effect of the EAL domain on TTSS-1 secretion of SipA. To extrapolate, we conclude that the c-di-GMP metabolizing proteins inhibit not only the secretion of SipA, but several, if not all, TSSS-1 effector proteins in order to contribute to the inhibition of the invasion phenotype. This issue is currently under investigation. However, we also have to note that inhibition of TTSS-1 secretion most likely does not require c-di-GMP (although a contribution of the c-di-GMP binding I-site in the GGDEF domain [36] cannot be excluded). Thus inhibition of the invasion phenotype by c-di-GMP signaling must be mediated by another pathway.

### Effect of double mutants on the invasion phenotype

In order to investigate whether the mutants work in the same pathway or contribute to the same c-di-GMP pool, we constructed double mutants for all GG(D/E)EF and EAL domain proteins, which showed a statistically significant conventional phenotype. Subsequently, we investigated the invasion phenotype in comparison with the single mutants.

**Figure 4:**
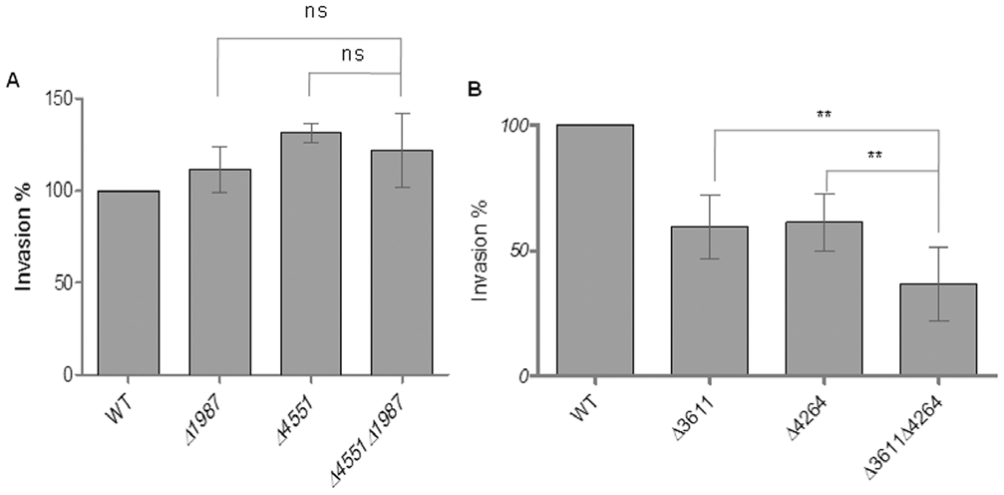
Effect of double mutants of GG(D/E)EF or EAL domain proteins on invasion of *S. typhimurium* into HT-29 cells. (A) Invasion is not significantly enhanced in the double mutant of the GG(D/E)EF domain proteins STM4551 and STM1987 as compared to the single mutants. (B) Invasion of the double mutant of the EAL proteins STM3611 and STM4264 into HT-29 cells is significantly decreased as compared to the single mutants. Experimental conditions as in Figure 1. WT = wild type *S. typhimurium* UMR1. Bars show mean ± standard deviation from at least 5 independent biological experiments performed in two technical replicates. **P<0.01 as compared with the corresponding single mutant.

The STM1987/STM4551 double mutant (121±15% of wild type UMR1) was not more invasive than the STM1987 and STM4551 single mutants (Fig. 4A) suggesting that there is an upper threshold of invasiveness, which is independent of c-di-GMP signaling.

Five EAL-domain proteins, STM0343, STM0468, STM2215, STM3611 and STM4264, positively contribute to invasion. Investigation of all combinations of double mutants for the invasion phenotype showed that the STM4264/STM3611 double mutant showed a 61±12% reduction in invasion (compared to wild type UMR1), a significant phenotype compared with the two single mutants (Fig. 4B). All the other double mutants did not show a significant alteration of the invasion phenotype as compared to the single mutants (Fig. S4). This data suggests that at least some of the mutants affect the same pathway(s).

### Identification of corresponding di-guanylate cyclases and phosphodiesterases

**Figure 5:**
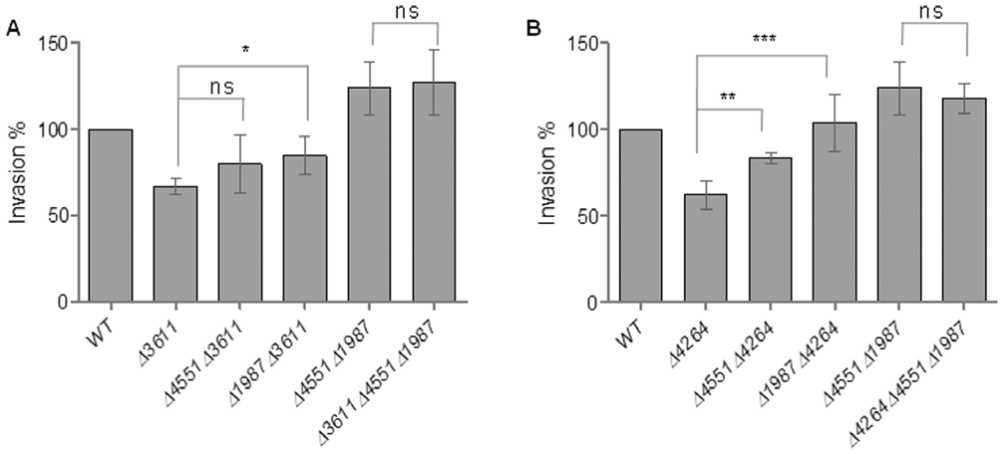
Corresponding di-guanylate cylcases and phosphodiesterases in the regulation of the invasion phenotype. (A) The decreased invasion phenotype of the STM3611 mutant is partially restored in the STM3611/STM1987 double mutants indicating that STM3611 has a minor effect on the degradation of the c-di-GMP produced by STM1987 that affects invasion. Invasion restauration occurs also in the STM3611/STM4551 double mutant, but a significant effect was not achieved. (B) The decreased invasion phenotype of the STM4264 mutant is restored to wild type levels in the STM4264/STM1987 double mutant indicating that STM4264 is the major phosphodiesterase degrading the c-di-GMP produced by STM1987. STM4551 deletion has a signficant, but less pronounced effect on the restauration of invasion in the STM4264 mutant. (A and B) Invasion of the STM3611 and STM4264 mutant is restored by the deletion of STM1987 and STM4551 indicating that STM1987 and STM4551 are the two major di-guanylate cyclases producing the c-di-GMP that inhibits invasion. Experimental conditions as in Figure 1. WT = wild type *S. typhimurium* UMR1. Bars show mean ± standard deviation from at least 4 independent biological experiments performed on two technical replicates. Statistical significance is indicated by *p<0.05, **p<0.01, ***p<0.001 as compared with STM4264 (A) and STM3611 mutant (B).

C-di-GMP metabolizing enzymes with opposing function often work in pairs [37]. C-di-GMP produced by a di-guanylate cyclase is degraded by a specific phosphodiesterase(s). Consequently, deletion of the corresponding di-guanylate cyclase in the background of a phosphodiesterase mutation should at least partially elevate the phenotype of the phosphodiesterase mutant. On the other hand, deletion of a di-guanylate cyclase, the c-di-GMP pool of which is not affected by the specific phosphodiesterase, should not alter the phenotype of the phosphodiesterase mutant. To this end, we compared the invasion rate of the STM3611 phosphodiesterase mutant when the di-guanylate cyclase(s) STM4551 and/or STM1987 were deleted. STM4551 and/or STM1987 were also deleted in the STM4264 mutant background. Indeed, when both di-guanylate cyclases were deleted in the STM3611 or STM4264 background a hyper-invasion phenotype comparable to the STM4551/STM1987 double mutant was observed (Fig. 5). This result suggests that these two di-guanylate cyclases produce the majority of the c-di-GMP inhibiting invasion. Most of the repressive effect of the STM4264 mutation was relieved when STM1987 was deleted, suggesting that STM4264 degrades mainly c-di-GMP produced by STM1987 and dedicated to invasion inhibition (Fig. 5B). Significant elevation of repression of invasion was also observed in the STM4551/STM4264 and STM1987/STM3611 double mutants, although the effect was less pronounced (Fig. 5A, B).

### Contribution of CsgD and the cellulose synthase BcsA to inhibition of the invasion phenotype

**Figure 6:**
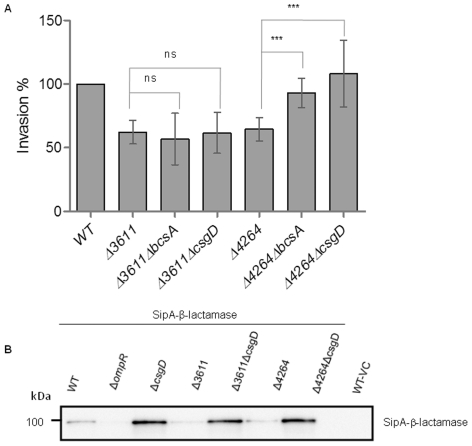
The EAL proteins STM3611 and STM 4264 affect invasion of *S. typhimurium* through different pathways. (A) Deletion of the gene encoding transcription regulator of rdar biofilm formation CsgD and the gene encoding the cellulose synthase BcsA restored invasion to wild type levels in the STM4264 mutant. Deletion of *csgD* or *bcsA* in the STM3611 mutant did not alter the invasion capability of STM3611. Experimental conditions as in Figure 1. WT = wild type *S. typhimurium* UMR1. Bars show mean ± standard deviation from at least 4 independent biological experiments performed on two technical replicates. Statistical significance is indicated by ***p<0.001. (B) Deletion of *csgD* in the STM4264 and STM3611 mutants restored secretion of SipA. Upregulation of SipA secretion was observed also when *csgD* was deleted in the wild type background, although no upregulated invasion has been observed in the *csgD* mutant (Fig. S5; [15]). SipA-β-lactamase indicates chromosomal fusion in respective strains. Detection of the SipA-β-lactamase fusion protein by western blot analysis using an anti-β-lactamase antibody. Strain *S. typhimurium* UMR1 with pBAD30 expressing β-lactamase in the periplasm (WT-VC) served as β-lactamase secretion control. WT = wild type *S. typhimurium* UMR1; Δ*ompR*, negative control; VC = vector control pBAD30.

Previously, we have shown that inhibition of invasion by high concentrations of c-di-GMP is partially mediated through the biofilm regulator CsgD and the cellulose synthase BcsA [15]. We wanted to verify the participation of CsgD and BcsA to invasion inhibition at physiologically elevated levels of c-di-GMP. Deletion of CsgD and BcsA in the STM4264 phosphodiesterase mutant restored invasion to wild type levels (Fig. 6A). In contrast, in the STM3611 mutant, deletion of CsgD and BcsA had no effect. This finding shows that c-di-GMP degraded by STM4264 is inhibiting invasion through CsgD and BcsA suggesting that CsgD and BcsA work downstream of STM4264. On the other hand, as deletion of CsgD and BcsA did not relieve the hypo-invasion phenotype of the STM3611 mutant, although CsgD contributes to relieve of TTSS-1 secretion in the STM3611 mutant (see below), c-di-GMP degraded by STM3611 affects invasion also downstream of CsgD and BcsA or in a parallel pathway.

Expression of CsgD inhibits secretion of the TTSS-1 effector protein SopE2 [15] and SipA (Fig. 6B). Enhanced SipA secretion in the *csgD* mutant can be complemented by plasmid-expressed CsgD (Fig. S5) Deletion of *csgD* in the STM3611 and STM4264 mutant background restored secretion of the TTSS-1 effector protein SipA (Fig. 6B). Collectively, these findings suggest that CsgD (at least partially) inhibits invasion in the STM4264 mutant through repression of secretion of SipA and other TTSS-1 effector proteins (our unpublished data; [15]). In the STM3611 mutant background, however, relieve of repression of TTSS-1 secretion by CsgD is not sufficient to restore invasion.

### Screening of GG(D/E)EF/EAL domain proteins for the cytokine induction phenotype

**Figure 7:**
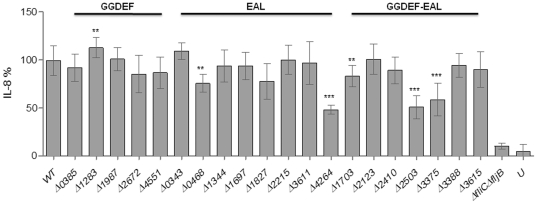
Individual GG(D/E)EF/EAL domain proteins affect *S. typhimurium* induced IL-8 production by the gastrointestinal cell line HT-29. Relative IL-8 production of HT-29 cells after incubation with individual GG(D/E)EF/EAL mutants. IL-8 production of the wild type (WT) *S. typhimurium* UMR1 was set 100%. Δ*fliC*Δ*fljB*, negative control, U = unstimulated cells. Strains were grown under invasion inducing conditions (standing culture, LB+0.3M NaCl) until O.D._600_ 0.6 and co-incubated with HT-29 cells for 1 h. IL-8 production of HT-29 cells was measured by ELISA. Bars show mean % ± standard deviation from at least three independent biological experiments performed in two technical replicates. Statistical significance is indicated by *P<0.05, **P<0.01, ***P<0.001 as compared with wild type *S. typhimurium* UMR1.

The pro-inflammatory cytokine interleukin-8, IL-8, is produced by epithelial cells, when infected with *S. typhimurium* [38], [39]. A well-studied model uses the gastrointestinal epithelial cell line HT-29 where induction of IL-8 is dependent on recognition of secreted monomeric flagellin by TLR5 [40]. High intracellular c-di-GMP levels in *S. typhimurium* prevented induction of IL-8 production in HT-29 cells [15], consequently we analyzed the role of the individual chromosomally expressed c-di-GMP metabolizing proteins on the ability to stimulate IL-8 production. 6 of 20 mutants showed a significant alteration of the stimulation of IL-8 production (Fig. 7).

Screening of the GG(D/E)EF domain protein deletion mutants for alteration of IL-8 production revealed a 13±7% rise in IL-8 production for the STM1283 mutant.

Screening of EAL deletion mutants demonstrated statistically significant effects for STM0468 and STM4264. Deletion of STM4264 led to a 61±5% reduction of IL-8 production compared to wild type UMR1, while the absence of STM0468 reduced the IL-8 response to 76±7% of the wild type level.

Screening of GG(D/E)EF-EAL protein mutants revealed an altered phenotype for the STM1703, STM2503 and STM3375 mutants. IL-8 production decreased to 83±7%, 59±8% and 47±12% of wild type levels was observed.

The groups of mutants which affect invasion and the IL-8 induction phenotype are distinct, but overlap (Fig. 1, Fig. 7). Three mutants affected the invasion and the IL-8 induction phenotype. The EAL mutants STM0468 and STM4264 showed reduced invasion and IL-8 induction, while the GGDEF mutant STM1283 enhanced IL-8 induction, but showed reduced invasion.

### Complementation of the cytokine induction phenotype of GG(D/E)EF/EAL mutants

**Figure 8:**
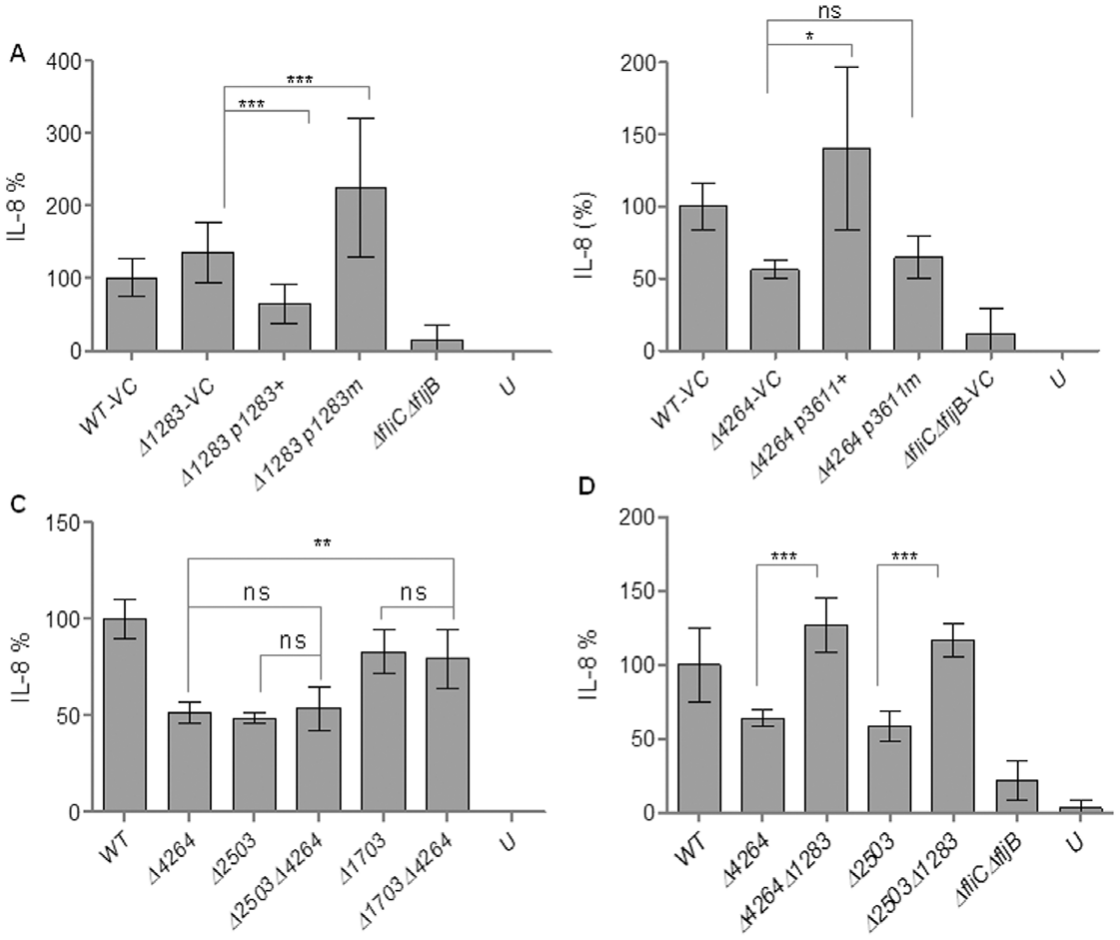
Complementation of the IL-8 induction phenotype of GG(D/E)EF/EAL protein mutants and double mutant analysis. (A) Complementation of the STM1283 mutant with STM1283 (p1283+) and STM1283_D425A_ (p1283m) demonstrates requirement of the di-guanylate cyclase activity of STM1283 for complementation of the IL-8 induction phenotype as the enhanced IL-8 induction rate exhibited by the STM1283 mutant was significantly reduced by complementation with wild type STM1283, but not with the non-functional mutant STM1283_D425A_ with the GGDEF motif altered to GGAEF. (B) Complementation of the STM4264 mutant with STM3611 (p3611+) and STM3611_K179A_ (p3611m) demonstrates requirement of the phosphodiesterase activity of STM4264 for complementation of the IL-8 induction phenotype. The reduced IL-8 induction rate exhibited by the STM4264 mutant was increase to wild type levels by complementation with the EAL-only protein STM3611, but not with the catalytically inactive mutant STM3611_K179A_. (C) No additive effect on the reduction of IL-8 production is observed when double mutants of STM2503/STM4264 and STM1703/STM4264 were compared to the respective single mutants. (D) Deletion of the gene encoding the phosphodiesterase STM4264 or the putative phosphodiesterase STM2503 has no effect on IL-8 expression in the STM1283 mutant background indicating that STM4264 and STM2503 degrade the c-di-GMP produced by the di-guanylate cyclase STM1283. WT = wild type *S. typhimurium* UMR1; VC = vector control pBAD30; Δ*fliC*Δ*fljB*, negative control, U = unstimulated HT-29 cells. Bars show mean ± standard deviation from at least three independent biological experiments performed in two technical replicates. Statistical significance is indicated by *P<0.05, **P<0.01, ***P<0.001 as compared with the respective single mutant.

To unambiguously couple the IL-8 induction phenotype of the mutations in GG(D/E)EF/EAL domain proteins and c-di-GMP signaling, we performed complementation and mutation studies. We complemented the STM1283 mutant with STM1283 cloned in the vector pBAD30 under the control of the arabinose-inducible promoter. Expression of STM1283 from the plasmid abolished the IL-8 hyper-production phenotype of the STM1283 mutant and lead to an IL-8 induction as low as 56±10% of the wild type (Fig. 8A). The STM1283 mutant was the only mutant in a predicted di-guanylate cyclase (see Fig. S1), which significantly affected IL-8 production. However, overexpression of the STM4551 di-guanylate cyclase in the STM1283 mutant equally downregulated IL-8 production (data not shown). To demonstrate the participation of c-di-GMP signaling to the phenotype, we mutated the GGDEF motif to GGAEF (mutant protein STM1283_D425A_) to abolish the predicted di-guanylate cyclase activity of STM1283. Indeed, expression of STM1283_D425A_ in the STM1283 mutant failed to reverse the IL-8 hyper-production phenotype of the STM1283 mutant (Fig. 8A). This finding suggests that STM1283 affects IL-8 induction through c-di-GMP signaling. However, we could not detect a rise in c-di-GMP concentration by HPLC analysis when STM1283 was overexpressed (Fig. S2). This finding suggests that STM1283 produces minor amounts of c-di-GMP, which work locally.

The most pronounced alteration of IL-8 induction was seen with the EAL mutants STM4264 and STM2503, 61±5% and 59±8% reduction compared to wild type levels, respectively. Overexpression of the phosphodiesterase STM3611, but not its catalytically inactive mutant STM3611_K179A_ restored wild type levels of IL-8 production in these mutants (Fig. 8B and data not shown). Consequently, the c-di-GMP specific phosphodiesterase activity of the two proteins is required for expression of the phenotype. Also complementation of other EAL or GG(D/E)EF-EAL protein mutants with a conventional phenotype in IL-8 secretion with STM3611 restored wild type levels of IL-8 production (Fig. S6). Collectively, these data demonstrate that c-di-GMP signaling regulates the IL-8 induction phenotype in *S. typhimurium*.

### Effect of double mutants on IL-8 production

To investigate whether the c-di-GMP metabolizing proteins that affected IL-8 production act in the same pathway or degrade an identical c-di-GMP pool, double mutants were constructed. The levels of IL-8 production, however, did not change in the double mutant STM2503 STM4264 compared to the STM4264 single mutant (Fig. 8C). Also in the other mutants no significant additive effect on the IL-8 production phenotype was observed (Fig. 8C, Fig. S7).

We hypothesized that STM1283 is the di-guanylate cyclase producing the c-di-GMP which leads to the inhibition of the IL-8 production phenotype once phosphodiesterases are absent. Indeed, deletion of STM4264 and STM2503 in the STM1283 mutant background retained IL-8 levels as in the STM1283 mutant (8D) suggesting that STM4264 and STM2503 degrade the c-di-GMP produced by STM1283 dedicated to inhibition of IL-8 induction.

### Effect of CsgD on IL-8 production in EAL mutants

**Figure 9:**
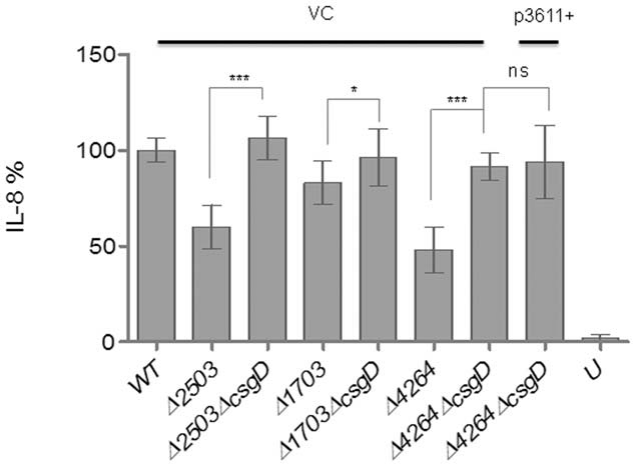
Impairment of IL-8 production by STM2503, STM1703 and STM4264 mutants is relieved by deletion of the biofilm regulator CsgD. IL-8 production of HT-29 cells co-incubated with phosphodiesterase EAL-domain proteins/*csgD* double mutants was monitored. Restoration of immunogenicity was observed when *csgD* was deleted in combination with EAL-domain proteins. Overexpression of the phosphodiesterase STM3611 (p3611+ = STM3611 in pLAFR3) in the STM4264/*csgD* double mutant provoked no additional IL-8 production. WT = wild type *S. typhimurium* UMR1; VC = vector control pLAFR3; U = unstimulated HT-29 cells. Bars show mean ± standard deviation from at least three independent biological experiments performed in two technical replicates. Statistical significance is indicated by *P<0.05, **P<0.01, ***P<0.001 as compared with the respective single mutant.

Our previous results have shown that deletion of *csgD* abolished the repressive effect of c-di-GMP on IL-8 induction [15]. Consequently, the effect of a c*sgD* deletion on the IL-8 induction phenotype in the individual phosphodiesterase mutants was investigated. When *csgD* was deleted in the STM1703, STM2503 and STM4264 mutants (Fig. 9), IL-8 production was restored to wild type levels revealing that c-di-GMP signaling affects *S. typhimurium* immunogenicity through CsgD.

### Identification of GG(D/E)EF/EAL-domain proteins involved in *in vivo* survival and cecum colonization of *S. typhimurium*

**Figure 10:**
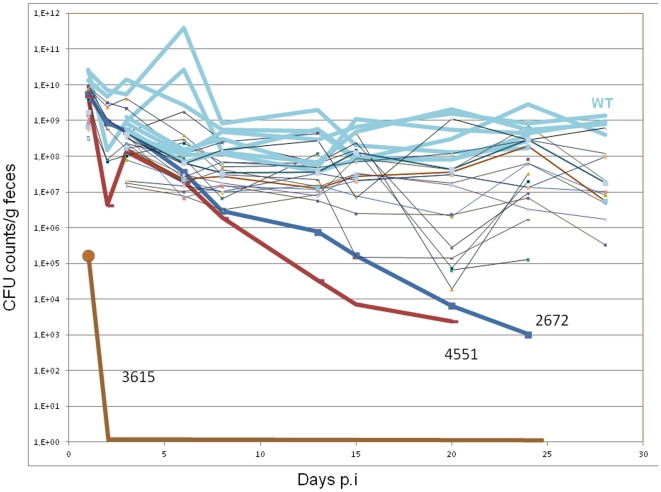
Presence of GG(D/E)EF/EAL mutants of *S. typhimurium* UMR1 in fecal pellets of streptomycin treated mice. Streptomycin treated mice were infected with groups of four strains (wild type and three GG(D/E)EF/EAL mutants). Clearance of mutants from murine intestine was evaluated by CFU counts per gram feces until 30 days after infection. Three of twenty mutants (STM2672, STM3615 and STM4551) were cleared early from the murine intestine. WT = *S. typhimurium* UMR1.

As c-di-GMP signaling is involved in the regulation of virulence phenotypes *in vitro*, we investigate the effect of c-di-GMP signaling in a virulence model *in vivo*. To test the GG(D/E)EF/EAL domain protein mutants *in vivo* we chose the streptomycin-treated mouse model [41] and studied colonization and survival in the murine intestine. Mutants were screened in competition experiments in groups of four (including the wild type) for colonization of the mouse intestinal tract which investigates a complex phenotype including survival, adherence, invasion of host cells and escape from host clearance. Monitoring colonization as viable bacterial counts in feces during 30 days revealed significant phenotypes for three GG(D/E)EF/EAL domain proteins. Mutants harboring the deletion of STM2672, STM3615 and STM4551 were attenuated in their colonization ability already from day 12 post-infection (Fig. 10). These results were confirmed in direct competition experiments with the wild type (data not shown). On the 30^th^ day post-infection the mice were sacrificed and the cecum content was analyzed. 9 of 20 mutants showed reduced cecum colonization of at least 100-fold, with the most significant decrease observed for the STM1987, STM2410, STM2672, STM3615 and STM4551 mutants (Fig. 10).

## Discussion

In this work, we demonstrated that individual GG(D/E)EF/EAL domain proteins of *S. typhimurium*, which are proven or predicted di-guanylate cyclases and c-di-GMP phosphodiesterases modulate invasion, secretion of the TTSS-1 effector protein SipA, IL-8 induction in epithelial cells and *in vivo* colonization. In combination with catalytic mutant analysis, this study confirms our previous finding, namely that c-di GMP signaling inhibits the acute virulence phenotypes invasion and IL-8 induction in *S. typhimurium* and thus reversely regulate virulence and biofilm formation [15].

Numerous GG(D/E)EF/EAL domain proteins affect the virulence phenotypes; 10 of 20 GG(D/E)EF/EAL domain proteins affected invasion and 6 of 20 IL-8 induction. Previously, c-di-GMP signaling was found to regulate biofilm formation in *S. typhimurium* [7], . In particular, eight GG(D/E)EF/EAL domain proteins regulate biofilm formation at ambient temperature on agar plates. The high number of GG(D/E)EF/EAL domain proteins affecting virulence phenotypes shows therefore that c-di-GMP signaling in *S. typhimurium* is not only relevant outside the host at low temperature in bacterial cell-cell interactions, but equally relevant at 37°C, the host temperature. In general, although c-di-GMP is not absolutely required for virulence [43], c-di-GMP is involved in regulation of virulence properties in plant and animal pathogens at different temperatures [28], [44], [45], [46], [47].

For six GGDEF/EAL domain protein mutants, STM0343, STM0468, STM1697, STM1283, STM2503 and STM3615, a phenotype was identified for the first time. Besides STM1283, all other proteins are EAL domain proteins and, besides STM1697, predicted c-di-GMP specific phosphodiesterases (Table 1). This finding highlights the important role of phosphodiesterases in the regulation of virulence phenotypes.

Two distinct groups of GG(D/E)EF/EAL domain proteins alter the invasion and IL-8 induction phenotype. In addition, also the phenotype *in vivo* colonization is regulated by a distinct group of GG(D/E)EF/EAL proteins. The concept of ‘group-specific’ behavior of c-di-GMP signaling proteins correlates with previous findings in *S. typhimurium* and other bacteria where distinct groups of c-di-GMP signaling proteins modulate biofilm formation, motility and virulence phenotypes [48] [29] [28]. It is consistent with the concept that certain c-di-GMP signaling pathways are dedicated to regulate specific cellular functions or the same cellular function on different levels and suggests a spatial and temporal compartmentalization of the signal and different effector proteins [49]. On the other hand, we also observed redundancy of function for some of the GG(D/E)EF/EAL proteins (Fig. 8; Fig. S4, S7).

**Figure 11:**
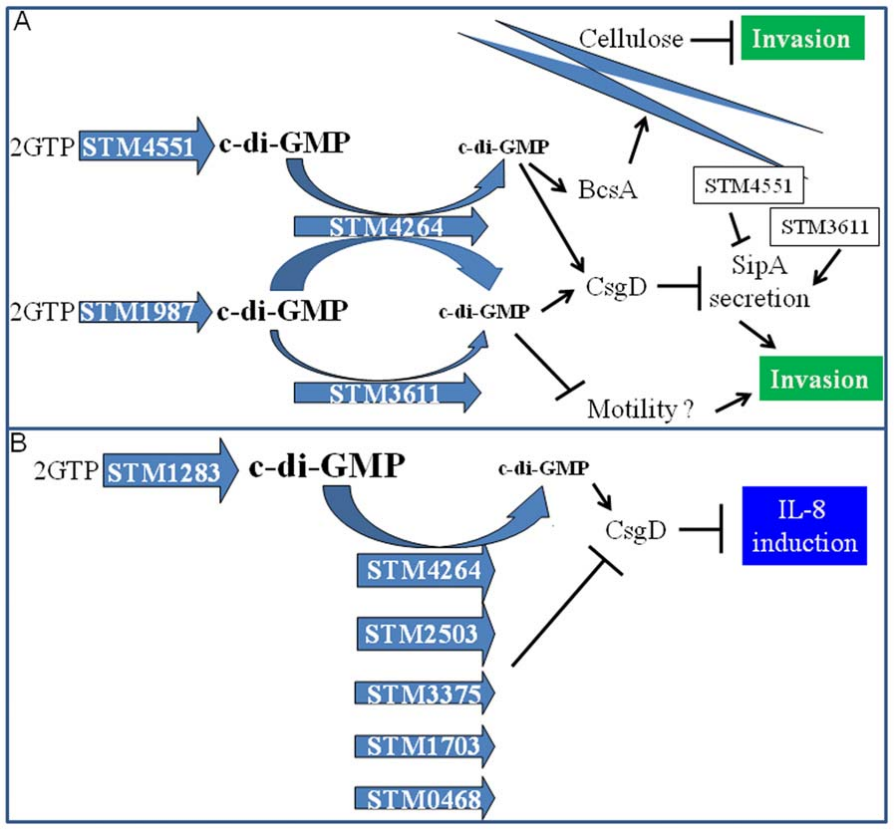
Working models of the regulatory networks of c-di-GMP signaling in *S. typhimurium* leading to suppression of invasion and IL-8 induction. (A) A model of the c-di-GMP signaling network for the suppression of the invasion phenotype by c-di-GMP signaling. Shown are di-guanylate cyclases and phosphodiesterases with the most pronounced effect on invasion. Activity of the di-guanylate cyclases STM1987 and STM4551 and the phosphodiesterases STM3611 and STM4262 create different c-di-GMP pools which subsequently affect target outputs. Upon elevated c-di-GMP, the invasion phenotype is negatively regulated by c-di-GMP signaling through the cellulose synthase BcsA and the biofilm regulator CsgD. CsgD inhibits secretion of TTSS-1 effector protein SipA. SipA secretion is affected by GGDEF and EAL domain proteins. Whether motility affects the invasion phenotype needs to be demonstrated. (B) Model of the c-di-GMP signaling network for the suppression of the IL-8 induction phenotype by c-di-GMP signaling. The c-di-GMP pool created by the di-guanylate cyclase STM1283 is degraded by the phosphodiesterases STM0468, STM1703, STM2503, STM3375 and STM4264 which are shown in the order of affection of the IL-8 induction phenotype. Upon elevation of c-di-GMP the resulting c-di-GMP pool is suggested to stimulate CsgD expression which subsequently represses IL-8 induction.

We found that the invasion phenotype is regulated on different levels by the c-di-GMP signaling network. The phosphodiesterases STM3611 and STM4264 upregulate invasion, but partially through different pathways. In the STM4264 mutant, inhibition of the secretion of the TTSS-1 effector protein SipA and expression of the biofilm regulator CsgD and the cellulose synthase BcsA contribute to down-regulation of invasion (Fig. 11A). Deletion of CsgD restores invasion and SipA secretion. Most likely CsgD transcriptionally regulates a TSSS-1 component upstream of SipA. We have previously shown that BcsA does not inhibit the secretion of the TSSS-1 effector protein SopE2 [15]. However, expression of the exopolysaccharide cellulose by BcsA might put a sterical hindrance to *Salmonella* to invade epithelial cells. Whether CsgD is required for cellulose expression under invasion conditions remains to be shown. In the STM3611 mutant, however, deletion of CsgD relieves repression of SipA secretion but does not restore invasion. Therefore, we conclude that in the STM3611 mutant background the c-di-GMP signaling network affects another function besides TSSS-1 secretion that contributes to inhibition of invasion (Fig. 11A). This conclusion is supported by the fact that TTSS-1 secretion is modulated by c-di-GMP metabolizing proteins, but does not require their catalytic activity, while invasion is clearly modulated by c-di-GMP signalling. The altered motility in the STM3611 background could affect the invasion of *S. typhimurium*. On the other hand, alterations in the IL-8 induction phenotype did not correlate with the c-di-GMP modulated motility phenotype of the respective mutants (Fig. 1; Fig. 7; [12]).

CsgD is a central component in c-di-GMP mediated reduction of IL-8 stimulation (Fig. 11B) and contributes to the inhibition of invasion. The mechanism of CsgD inhibiting IL-8 stimulation remains to be determined, but it has been shown previously that CsgD transcriptionally regulates components of the flagella regulon [50], [51]. One of these components might control the secretion of monomeric flagellin.

CsgD expression is predicted to be a major target of c-di-GMP metabolism under invasion conditions as it is in plate-grown biofilm [11]. Also VpsT, a *V. cholera* homologue of CsgD shows significantly enhanced expression upon increased c-di-GMP concentrations [27]. In addition, VpsT positively regulates biofilm formation via c-di-GMP binding [52]. Although CsgD is a major target of the c-di-GMP signaling network, CsgD does not interact with c-di-GMP [50].

The conventional view is that low c-di-GMP levels promote acute infection phenotypes. Unconventional phenotypes were observed for some GG(D/E)EF/EAL domain proteins. The GG(D/E)EF domain protein STM1283 and the GG(D/E)EF-EAL domain protein STM2123 stimulated invasion, an unconventional phenotype as both proteins are predicted or demonstrated di-guanylate cyclases. At the same time though, the STM1283 mutant showed stimulation of IL-8 production (this work) and overexpression stimulated cellulose production in minimal medium [42] in concordance with STM1283 working as a di-guanylate cyclase. Previously, STM2123 was shown to be a di-guanylate cyclase stimulating the expression of CsgD in plate-grown *S. typhimurium* cells [11]. Unconventional phenotypes have been observed when GG(D/E)EF and/or EAL domains do not function as di-guanylate cyclases or c-di-GMP dependent phosphodiesterases [13], [44], [53], but affect a phenotype through e.g. c-di-GMP binding [53]. Binding of c-di-GMP to the I-site of GG(D/E)EF domains or an EAL domain affected biofilm formation in an unconventional fashion [54]. In addition, invasion and IL-8 production are already complex phenotypes, consisting of several steps. For example, effective invasion does not only require secretion of TTSS-1 effector components into host cells, but also adhesion to host cells [55], [56]. Secretion is usually stimulated by low c-di-GMP levels [27], [46], while adhesion is stimulated by high c-di-GMP levels [57].

Some of the GG(D/E)EF/EAL domain proteins identified in this study do not affect invasion through c-di-GMP signaling. Complementation studies showed that STM0468 affects invasion not through the EAL domain. Also, STM1697 lacks signature amino acids for c-di-GMP dependent phosphodiesterase activity and is, as STM1344 [13], to which it is highly similar most probably not directly involved in c-di-GMP metabolism and binding. In addition, although the STM3375 mutant phenotype is complemented by phosphodiesterase activity, the *E. coli* homologue of STM3375 affects biofilm formation through directing sRNAs for degradation [58].

Although we observed quite pronounced effects of GG(D/E)EF/EAL domain proteins on the invasion and IL-8 induction phenotype, we did not see a direct correlation with the colonization phenotype observed in the *in vivo* infection model. Actually, only mutant STM4551 had a cell culture and *in vivo* colonization phenotype (Fig. 1; Fig. 10). Indeed, although motility and chemotaxis has been implicated to play a role in *in vivo* colonization in the mouse model [59], [60], a role for flagellin secretion has only been defined for bovine colitis [61].

In summary, we have identified complex c-di-GMP signaling networks affecting virulence. The next step is then to elucidate the molecular mechanisms of inhibition of virulence phenotypes by c-di-GMP signaling.

## Materials and Methods

### Ethics Statement

All animals were handled in strict accordance with good animal practice as defined by the Bundesamt fur Veterinärwesen BVET and/or the local animal welfare bodies (Kantonales Veterinäramt Zürich). All animal work was approved by the appropriate committee (Kantonales Veterinäramt Zürich, Zürich, Switzerland, license number 201/2007).

### Bacterial strains, plasmids, and growth conditions

The bacterial strains and plasmids used in this study are listed in Table S1. For genetic manipulations, *Escherichia coli* and *S. typhimurium* were grown on Luria-Bertani (LB) agar plates or alternatively in LB liquid cultures supplemented with the appropriate antibiotics at 37°C overnight. To induce the invasion phenotype, *S. typhimurium* was grown overnight in LB+0.3M NaCl standing culture, diluted 1∶100 in new medium and grown until O.D. 0.6. The antibiotics used were ampicillin (100 µg ml^−1^), tetracycline (20 µg ml^−1^), kanamycin (30 µg ml^−1^) and chloramphenicol (20 µg ml^−1^). For induction of genes, 0.1% arabinose or 1 mM isopropyl-β-d-thiogalactopyranoside (IPTG) was used, if appropriate.

### Construction of mutants

Chromosomal mutations were generated using the Datsenko and Wanner method (Datsenko and Wanner, 2000). In general, entire open reading frames except 40 nucleotides at the beginning and at the end of the gene were replaced by either a kanamycin or chloramphenicol resistance marker. The *kan* or *cm* gene was PCR-amplified from pKD4 or pKD3, respectively, and electroporated into *S. typhimurium* ATCC 14028 carrying pKD46. Recovered colonies were purified at least twice on LB medium containing the corresponding antibiotics. If required, the resistance cassette was removed with the help of pCP20.

Phage transduction of mutant alleles into a novel strain background was carried out with phage P22 HT105/1 *int-201*. Transductants were colony purified twice on LB agar plates containing EGTA (10 mM) and appropriate antibiotics. All constructed mutants were verified by PCR with control primers located in the genes flanking the deleted open reading frame (Table S2).

pBAD30-derived plasmids from *E. coli* DH5α were passed through the restriction-deficient strain *S. typhimurium* SH9312 before electroporation into *S. typhimurium* UMR1 (ATCC14028-1s Nal^r^) derivatives. Triparental mating was conducted to introduce the plasmid pLAFR3 or its derivative pRGS3 (pLAFR3::STM3611) into *S. typhimurium* UMR1 derivatives. More specifically donor, cells containing helper-plasmid pRK2013 and recipient cells were mixed at a 1∶1∶1 ratio in 50 µL of LB. Mating was conducted on an LB plate incubated at 37°C overnight. At the end of incubation, cells were scrapped from the plate, resuspended in 1 ml of LB and plated at appropriate dilutions on selective medium containing tetracycline (20 µg ml^−1^) and the antibiotic selecting for the recipient.

### Plasmid construction

*Stm4551* and *stm1283* were amplified from *S. typhimurium* UMR1 using primer pair 4551-fw-SacI/4551-rev-6his-HindIII and primer pair 1283-fw-SacI/1283-rev-6his-HindIII respectively. Resulting PCR products were digested with restriction endonucleases *Sac*I and *Hin*dIII, and ligated with the *Sac*I/*Hin*dIII-restricted vector pBAD30. The inserted DNA sequence of plasmids pBAD30-4551 and pBAD30-1283 was confirmed by DNA sequencing [62].

### Construction of plasmids encoding mutated versions of STM1283, STM3611 and STM4551

To generate a mutation in the GGDEF motif of STM1283, the mutagenic oligos new-GGAEF-1283-fw and new-GGAEF-1283-rev were designed. The quick change mutagenesis kit (Agilent Technologies) was used according to the manufacturer's protocol with minor modifications. The resulting plasmid pBAD30-1283Mut coded for STM1283 with the GGAEF motif. Similarly, to create the mutation K179A in STM3611, the quick change mutagenesis kit was applied using oligos K179A forw and K179A Rev with plasmid pRGS1 as a template to obtain plasmid pBAD30::3611_K179A_.The mutation of the GGEEF motif of STM4551 to GGAEF was achieved by overlapping PCR. In brief, two overlapping fragments of *stm4551* were amplified using primer pair 4551-GGAEF-fw/4551-rev-6his-HindIII and primer pair 4551-fw-SacI/4551-GGAEF-rev respectively. These two overlapping PCR fragments were annealed in a second round of PCR and amplified using the outer primers 4551-fw-SacI and 4551-rev-6his-HindIII. The resulting PCR product was subjected to restriction with *Sac*I and *Hin*dIII and ligated into the *Sac*I/*Hin*dIII-restricted vector pBAD30.

All created mutations were confirmed by DNA sequencing. Functionality of cloned wild-type and mutated proteins was tested by investigating their effect on rdar morphotype expression, cellulose production and/or motility as described [12].

### Cell culture

The human epithelial cell line HT-29 (ATCC HTB 38, colon, colorectal adenocarcinoma) was grown to confluence in 24-well plates in RPMI-1640 medium (Life Technologies) supplemented with 25 mM Hepes, 2 mM l-glutamine, and 10% fetal calf serum (Sigma/Aldrich) at 37°C in 5% CO_2_.

### Invasion assay

Bacteria were diluted in RPMI-1640 medium and subsequently seeded on confluent HT-29 cells grown in 24-well plates at a multiplicity of infection of 1.7, which corresponds to 10^7^ cfu/ml. One hour postinfection, supernatant was removed and RPMI-1640 medium containing gentamicin at a final concentration of 100 µg/mL was added to the cells for 1 h to kill remaining extracellular bacteria. Cells were gently washed twice with PBS and disrupted with 1% Triton X-100 (Sigma Chemical). The number of intracellular bacteria was determined by colony forming units (CFU) counts of viable bacteria. An Δ*ompR* mutant was used as a negative control in all assays. OmpR is an activator of expression of the TSSS-1 activator HilA [63]. The invasion rate of a strain/mutant is defined as (CFU recovered inside cells after 1 h/CFU at time of inoculation)*100. The relative invasion rate is defined as (invasion rate of mutant/invasion rate of wild type)*100. Presented results are based on at least three biological replicates consisting of four technical replicates each.

### Stimulation of human epithelial cells

HT-29 cells were cultured in 24-well plates in RPMI-1640 medium. After a change of the medium, confluent layers of HT-29 cells were infected with respective bacterial strains grown under invasion inducing conditions at a MOI of 1.7, which corresponds to 10^7^ cfu/ml. Supernatants were collected after 1 h, centrifuged and analysed for production of IL-8 (IL-8 Elipair, BioSite).

### TTSS-1 secretion assay

Secretion of the TTSS-1 effector protein SipA was assayed as described [35]. Bacteria were precultured in LB liquid medium at 37°C overnight with appropriate antibiotics. The overnight culture was diluted 1∶33 in LB and grown with aeration at 37°C for approximately 3 h. 1 ml culture was removed at an OD_600_ = 0.7 and bacteria were spun down by centrifugation for 10 min at 14000 *g*. Secreted proteins were precipitated from the supernatant using 10% trichloroacetic acid (TCA) end concentration. Bacterial cells and the supernatant were subsequently analyzed for expression of SipA by Western blot.

### HPLC

Detection and quantification of c-di-GMP was performed exactly as described previously [15], [64].

### SDS-PAGE and Western blot

Proteins were run on a 6% SDS-PAGE gel and electro-transferred onto a PVDF membrane (Millipore Corp.) at 120 mA for 4 h. Membranes were blocked using 5% BSA and 5% non-fat dry milk in TBST (20 mM Tris at pH 7.5, 150 mM NaCl and 0.05% Tween-20) overnight. A rabbit anti-β-lactamase antibody (AB 3738, Millipore) was used as a primary antibody at a 1∶3000 dilution and anti-rabbit IgG (Jackson Immunoresearch) at a 1∶5000 dilution was used as secondary antibody. After washing, binding was detected using the ECL light detection reagent (Roche). Visualization of the detected bands was performed using FUJI LAS1000-plus chemiluminescence imaging system (Fuji, Stamford, CT, USA).

### Animal experiment

The ability of *S. typhimurium* strains to colonize and persist in the intestinal tract was analyzed using streptomycin-pretreated mice as a model for *Salmonella*-induced colitis [41]. All aspects of animal procedures were approved by Swiss authorities and performed according to the legal requirements. Sex- and age-matched specified pathogen free (SPF) 129Sv/Ev (Elévage Janvier) mice were held under barrier conditions at the Rodent Centre, Swiss Institute of Technology Zurich, Zurich, Switzerland. Infections were performed in individually ventilated cages (Tecniplast) as described [65]. Briefly, mice were pre-treated by gavage with 20 mg of streptomycin. Co-infection groups of 4 bacterial strains per 5 mice were prepared. Co-infection groups were consisting of streptomycin-resistant wild-type plus 3 mutant strains each carrying either ampicillin, kanamycin or tetracycline resistance along with the streptomycin resistance. For infection, bacteria were grown under invasion inducing conditions, washed twice in ice-cold PBS and suspended in cold PBS (5×10^7^ cfu each strain per 50 µl). 24 h after streptomycin pretreatment, the mice were intragastrically inoculated with the co-infection bacterial mix. Fresh fecal pellets were collected from individual mice aseptically every second day, starting on the first day after infection and for a period of 28 days. Fecal weight was determined and feces were suspended in PBS. Serial dilutions for plating were made in PBS and plated on MacConkey agar plates as described [41] for bacterial enumeration. Wild-type loads were determined by plating on medium containing 100 µg ml^−1^ streptomycin and co-infections with mutants were evaluated by replica-plating on medium containing appropriate antibiotics (ampicillin 100 µg/ml, kanamycin 50 µg/ml, tetracycline 20 µg/ml). 30 days after infection, the mice were sacrificed by cervical dislocation and the bacterial content in the cecum was determined as CFU counts per gram feces.

### Statistical analysis

Invasion and IL-8 data were compared using paired t-test. All the tests were two sided and values of p<0.05 were considered statistically significant. Animal experiment data were statistically analyzed by using the exact Mann-Whitney U Test. Graphs were created and statistical tests performed by using GraphPad Prism 4 version 4.03 (GraphPad Software).

## Supporting Information

Figure S1 **Classification of GGDEF and EAL domains of** ***S. typhimurium*** **.** (A) Classification of GGDEF domains. Class 1 GGDEF domains contain the GG(D/E)EF motif involved in substrate binding and catalysis and other signature amino acid residues. Class 1 GGDEF domains are predicted or proven di-guanylate cyclase (Table 1). Class 2 GGDEF domains do not contain the GG(D/E)EF motif and most other signature residues. Class 2 GGDEF domains are not predicted to possess di-guanylate cyclase activity. In green, GG(D/E)EF motif; in red, I-site, allosteric binding site for c-di-GMP for product inhibition [36]. (B) Classification of EAL domains. Class 1 EAL domains possess all highly conserved signature motifs [10], [31], [32] and are predicted or proven c-di-GMP specific phosphodiesterases. Class 2 EAL domains lack conservation of loop 6 and possess a potentially activatable catalytic function. Class 3 EAl domains lack catalytic activity. Colored residues indicate amino acids involved in catalysis, substrate and Mg^2+^ binding.
(TIF)

Figure S2 **C-di-GMP levels of strains overexpressing GG(D/E)EF domain proteins under invasion conditions.** Significantly higher c-di-GMP levels were observed when overexpressing the GGDEF domain protein STM4551 in the STM4551 mutant in comparison to wild type S. typhimurium UMR1 (WT), while overexpression of the catalytic mutant protein STM4551_E267A_ did not change the c-di-GMP levels. No change in c-di-GMP level was observed by overexpression of the GGDEF domain protein STM1283 and mutant protein STM1283_D425A_. Strains were grown under invasion inducing conditions (standing culture, LB+0.3M NaCl) until O.D.600 0.6. Bars show mean ± standard deviation from two independent biological experiments. VC = vector control pBAD30.
(TIF)

Figure S3 **Complementation of the invasion phenotype of putative phosphodiesterase mutants with catalytically inactive STM3611_K179A_.** The reduced invasion rate of EAL domain protein mutants of *S. typhimurium* was not restored to wild type level by complementation with a catalytically inactive mutant of STM3611, STM3611_K179A_, in plasmid pBAD30. WT = wild type *S. typhimurium* UMR1. VC = vector control pBAD30; p3611m = STM3611_K179A_ in plasmid pBAD30. Experimental conditions as in Figure 1. Bars show mean ± standard deviation from two independent biological experiments performed in two technical replicates.
(TIF)

Figure S4 **Analysis of the effect of double mutants in GG(D/E)EF/EAL domain proteins on invasion of** ***S. typhimurium*** **UMR1 into the HT-29 epithelial cell line.** (A–F) Invasion assay for double mutants of EAL domain proteins which previously showed significant downregulation of invasion. No statistically significant additive effect of the double deletion mutants was observed as compared to the respective single mutants. Experimental design and evaluation as in Figure 1. WT = wild type *S. typhimurium* UMR1. Bars show mean ± standard deviation from at least five independent biological experiments performed in two technical replicates.
(TIF)

Figure S5 **Complementation of the SipA secretion phenotype of the** ***csgD*** **mutant.** Enhanced secretion of the SipA-β-lactamase fusion protein in the *csgD* mutant is restored to wild type levels by expression of CsgD from plasmid pBAD30. Detection of the SipA-β-lactamase fusion protein by western blot analysis using an anti-β-lactamase antibody. Strain *S. typhimurium* UMR1 with pBAD30 expressing β-lactamase in the periplasm (WT-VC) served as β-lactamase secretion control. WT = wild type *S. typhimurium* UMR1; Δ*ompR*, negative control; VC = vector control pBAD30.
(TIF)

Figure S6 **Complementation of the IL-8 production phenotype of putative phosphodiesterase mutants.** Reduced IL-8 production of the EAL protein mutants STM2503, STM4264, STM1703, STM3375 and STM0468 is restored when the EAL-only domain phosphodiesterase STM3611 is expressed from plasmid pRGS3 (p3611). WT = wild type *S. typhimurium* UMR1; VC = vector control pLAFR3; U = unstimulated HT-29 cells. Bars show mean % ± standard deviation from at least three independent biological experiments performed in two technical replicates. Statistical significance is indicated by *P<0.05, **P<0.01, ***P<0.001 as compared with the corresponding vector control.
(TIF)

Figure S7 **Effect of double mutants in GG(D/E)EF/EAL domain proteins on IL-8 induction in HT-29 cells by** ***S. typhimurium*** **UMR1.** IL-8 induction assay for double mutants of GG(D/E)EF/EAL domain proteins, which previously showed significant downregulation of IL-8. No statistically significant additive effect of the double deletion mutants as compared to the respective single mutants was observed. Experimental design and evaluation as in Figure 7. WT = wild type *S. typhimurium* UMR1. Bars show mean ± standard deviation from four independent biological experiments performed in two technical replicates.
(TIF)

Table S1 Strains and plasmids used in this study.
(DOCX)

Table S2 Primers used in this study.
(DOCX)
