# Supplementary figures and images for: Complex c-di-GMP Signaling Networks Mediate Transition between Virulence Properties and Biofilm Formation in Salmonella enterica Serovar Typhimurium
Source: PLoS One. 2011 Dec 2;6(12):e28351. doi: 10.1371/journal.pone.0028351 (PMC3229569; doi:10.1371/journal.pone.0028351)

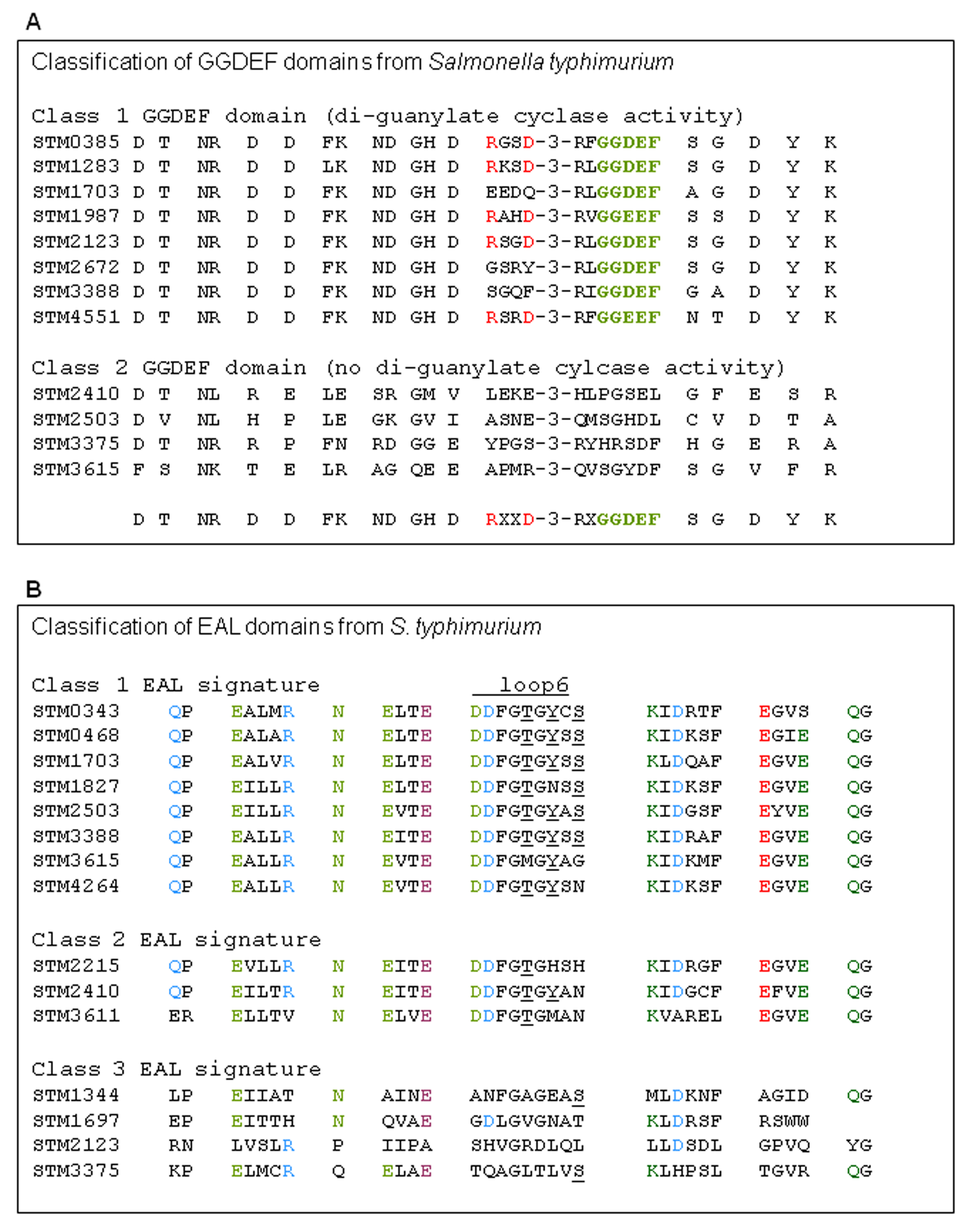

Supplement: Figure S1 — Classification of GGDEF and EAL domains of S. typhimurium . (A) Classification of GGDEF domains. Class 1 GGDEF domains contain the GG(D/E)EF motif involved in substrate binding and catalysis and other signature amino acid residues. Class 1 GGDEF domains are predicted or proven di-guanylate cyclase (Table 1). Class 2 GGDEF domains do not contain the GG(D/E)EF motif and most other signature residues. Class 2 GGDEF domains are not predicted to possess di-guanylate cyclase activity. In green, GG(D/E)EF motif; in red, I-site, allosteric binding site for c-di-GMP for product inhibition [36]. (B) Classification of EAL domains. Class 1 EAL domains possess all highly conserved signature motifs [10], [31], [32] and are predicted or proven c-di-GMP specific phosphodiesterases. Class 2 EAL domains lack conservation of loop 6 and possess a potentially activatable catalytic function. Class 3 EAl domains lack catalytic activity. Colored residues indicate amino acids involved in catalysis, substrate and Mg2+ binding. (TIF) [file pone.0028351.s001.tif]

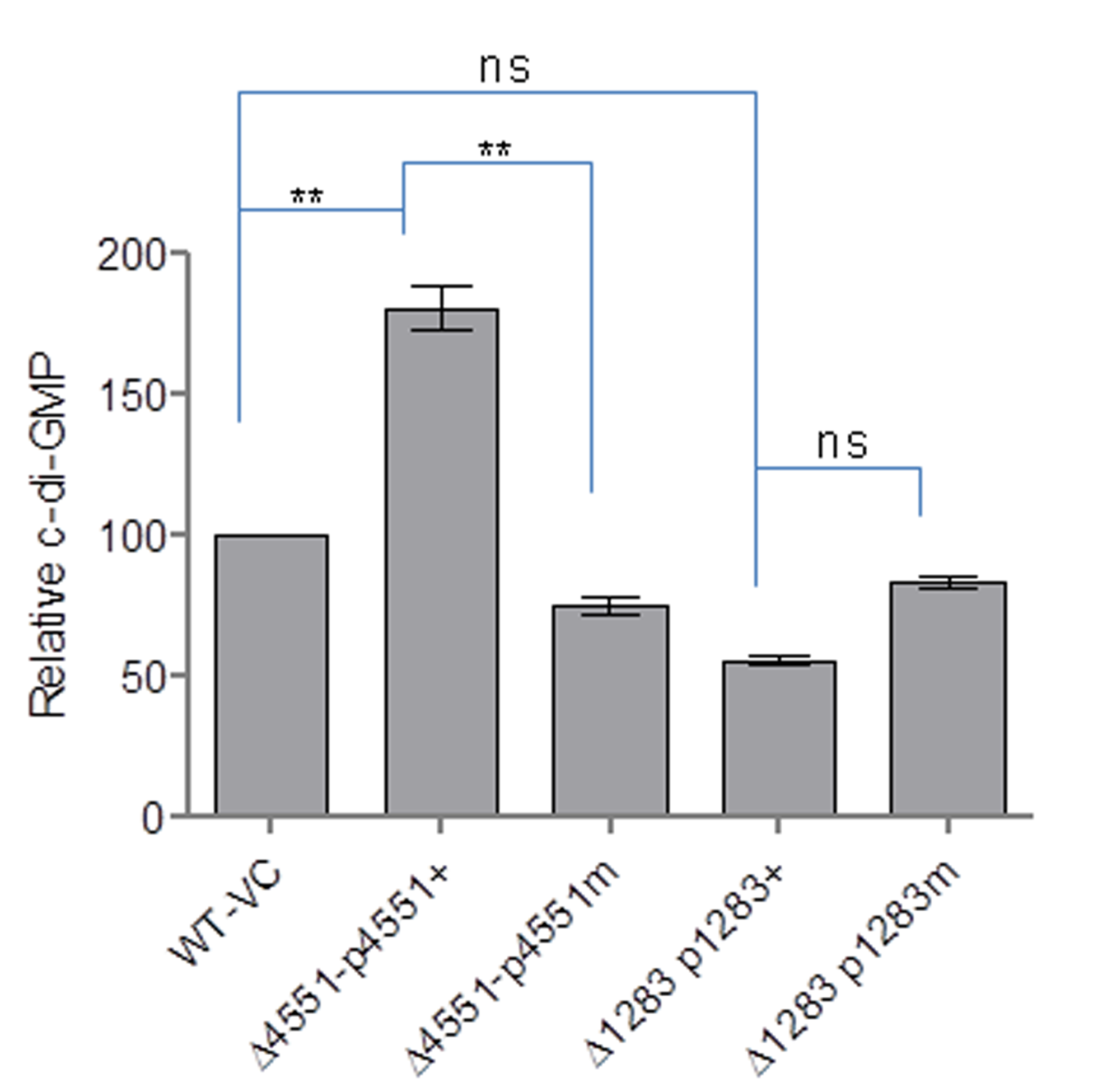

Supplement: Figure S2 — C-di-GMP levels of strains overexpressing GG(D/E)EF domain proteins under invasion conditions. Significantly higher c-di-GMP levels were observed when overexpressing the GGDEF domain protein STM4551 in the STM4551 mutant in comparison to wild type S. typhimurium UMR1 (WT), while overexpression of the catalytic mutant protein STM4551E267A did not change the c-di-GMP levels. No change in c-di-GMP level was observed by overexpression of the GGDEF domain protein STM1283 and mutant protein STM1283D425A. Strains were grown under invasion inducing conditions (standing culture, LB+0.3M NaCl) until O.D.600 0.6. Bars show mean ± standard deviation from two independent biological experiments. VC = vector control pBAD30. (TIF) [file pone.0028351.s002.tif]

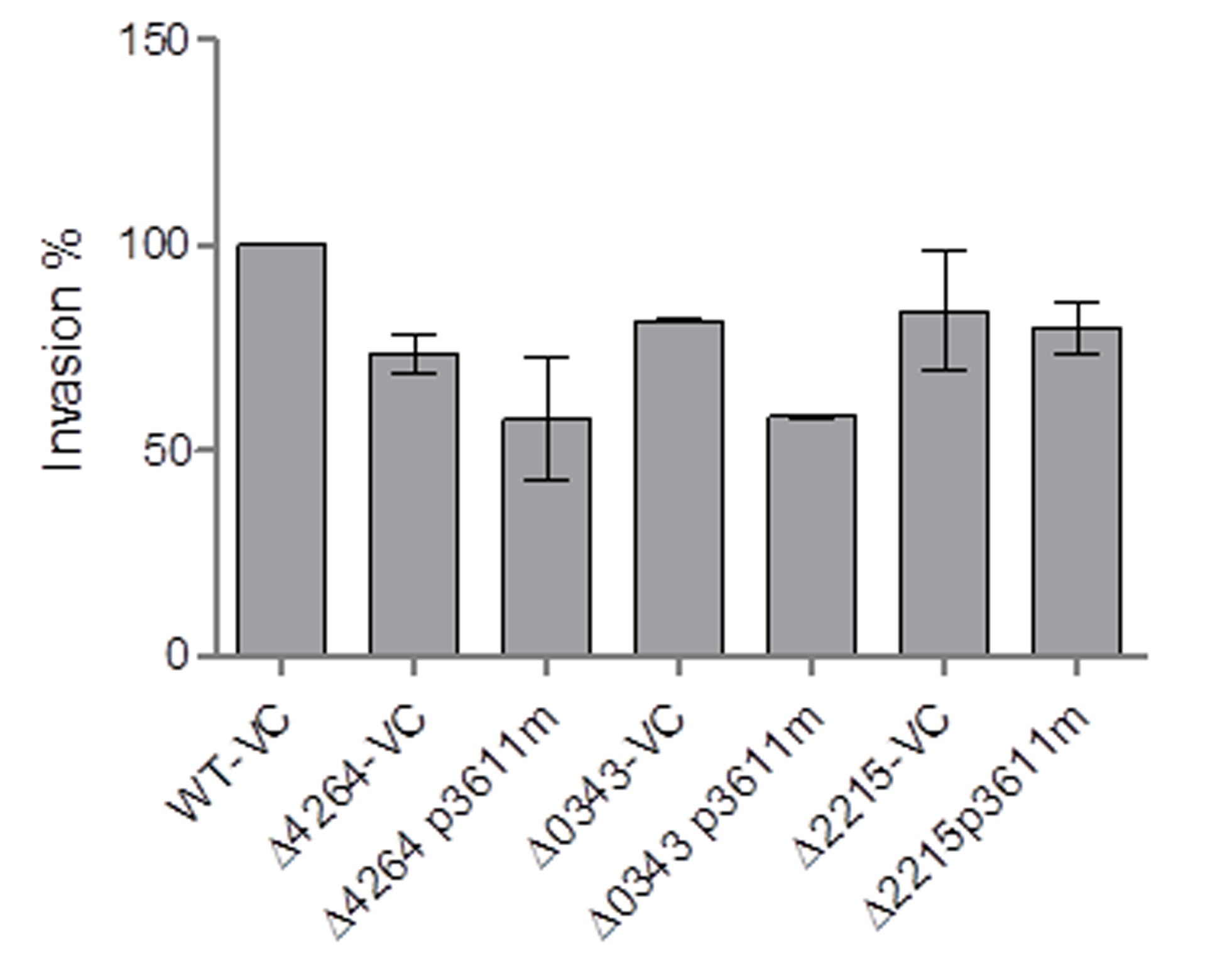

Supplement: Figure S3 — Complementation of the invasion phenotype of putative phosphodiesterase mutants with catalytically inactive STM3611K179A. The reduced invasion rate of EAL domain protein mutants of S. typhimurium was not restored to wild type level by complementation with a catalytically inactive mutant of STM3611, STM3611K179A, in plasmid pBAD30. WT = wild type S. typhimurium UMR1. VC = vector control pBAD30; p3611m = STM3611K179A in plasmid pBAD30. Experimental conditions as in Figure 1. Bars show mean ± standard deviation from two independent biological experiments performed in two technical replicates. (TIF) [file pone.0028351.s003.tif]

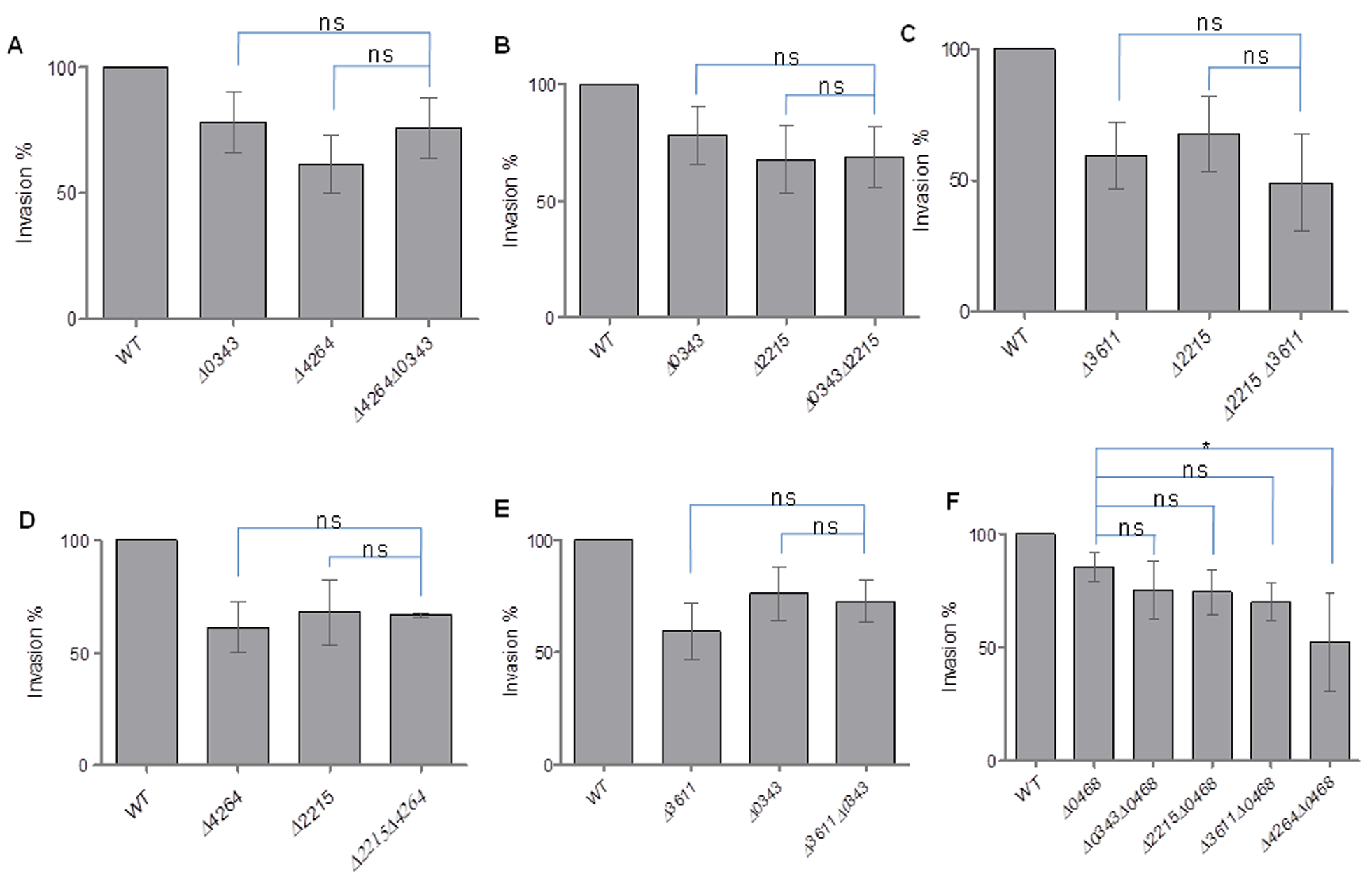

Supplement: Figure S4 — Analysis of the effect of double mutants in GG(D/E)EF/EAL domain proteins on invasion of S. typhimurium UMR1 into the HT-29 epithelial cell line. (A–F) Invasion assay for double mutants of EAL domain proteins which previously showed significant downregulation of invasion. No statistically significant additive effect of the double deletion mutants was observed as compared to the respective single mutants. Experimental design and evaluation as in Figure 1. WT = wild type S. typhimurium UMR1. Bars show mean ± standard deviation from at least five independent biological experiments performed in two technical replicates. (TIF) [file pone.0028351.s004.tif]

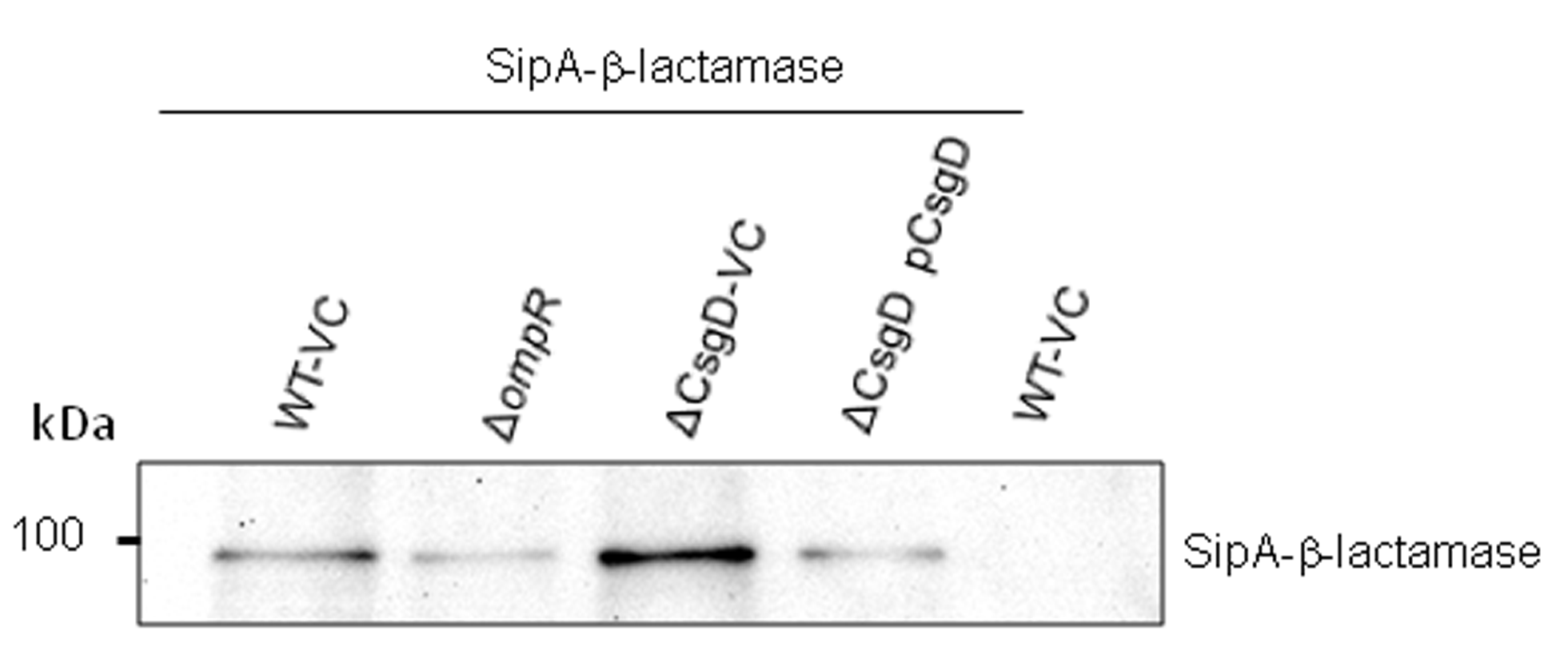

Supplement: Figure S5 — Complementation of the SipA secretion phenotype of the csgD mutant. Enhanced secretion of the SipA-β-lactamase fusion protein in the csgD mutant is restored to wild type levels by expression of CsgD from plasmid pBAD30. Detection of the SipA-β-lactamase fusion protein by western blot analysis using an anti-β-lactamase antibody. Strain S. typhimurium UMR1 with pBAD30 expressing β-lactamase in the periplasm (WT-VC) served as β-lactamase secretion control. WT = wild type S. typhimurium UMR1; ΔompR, negative control; VC = vector control pBAD30. (TIF) [file pone.0028351.s005.tif]

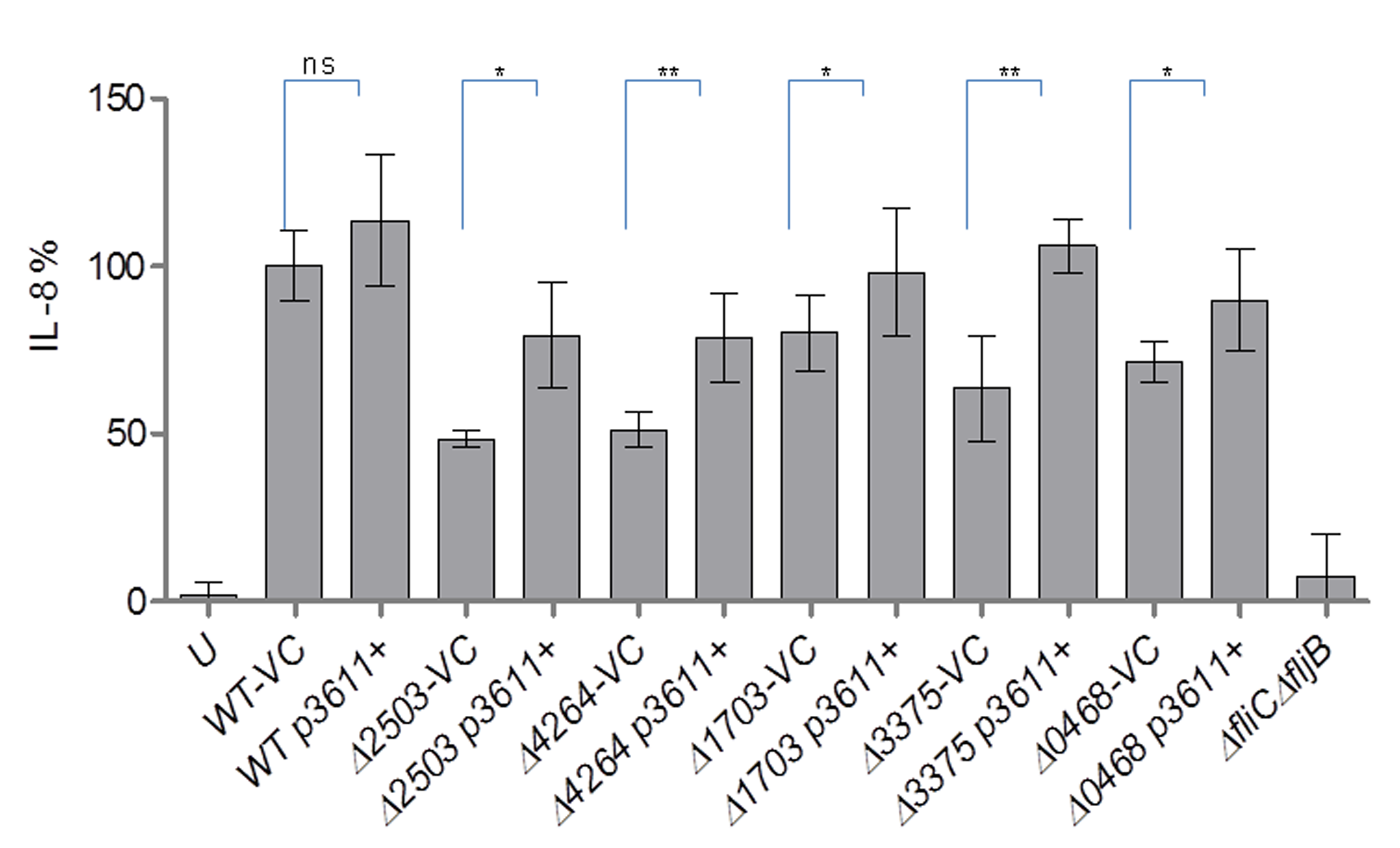

Supplement: Figure S6 — Complementation of the IL-8 production phenotype of putative phosphodiesterase mutants. Reduced IL-8 production of the EAL protein mutants STM2503, STM4264, STM1703, STM3375 and STM0468 is restored when the EAL-only domain phosphodiesterase STM3611 is expressed from plasmid pRGS3 (p3611). WT = wild type S. typhimurium UMR1; VC = vector control pLAFR3; U = unstimulated HT-29 cells. Bars show mean % ± standard deviation from at least three independent biological experiments performed in two technical replicates. Statistical significance is indicated by *P<0.05, **P<0.01, ***P<0.001 as compared with the corresponding vector control. (TIF) [file pone.0028351.s006.tif]

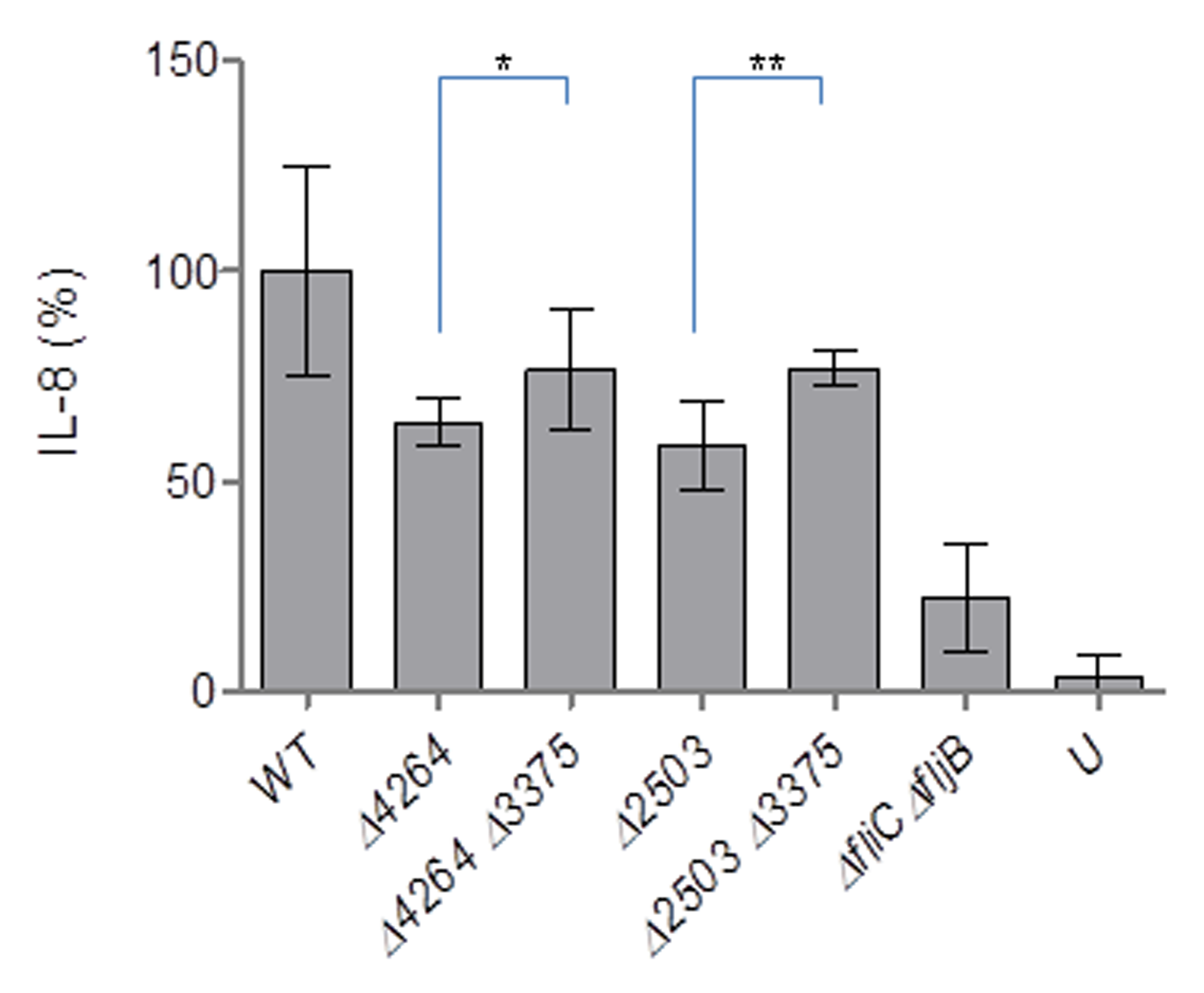

Supplement: Figure S7 — Effect of double mutants in GG(D/E)EF/EAL domain proteins on IL-8 induction in HT-29 cells by S. typhimurium UMR1. IL-8 induction assay for double mutants of GG(D/E)EF/EAL domain proteins, which previously showed significant downregulation of IL-8. No statistically significant additive effect of the double deletion mutants as compared to the respective single mutants was observed. Experimental design and evaluation as in Figure 7. WT = wild type S. typhimurium UMR1. Bars show mean ± standard deviation from four independent biological experiments performed in two technical replicates. (TIF) [file pone.0028351.s007.tif]
