# Supplementary material for: Complex c-di-GMP Signaling Networks Mediate Transition between Virulence Properties and Biofilm Formation in Salmonella enterica Serovar Typhimurium
Source: PLoS One. 2011 Dec 2;6(12):e28351. doi: 10.1371/journal.pone.0028351 (PMC3229569; doi:10.1371/journal.pone.0028351)
Supplement: Table S1 — Strains and plasmids used in this study. (DOCX) [file pone.0028351.s008.docx]

**Table S1**. Strains and plasmids used in this study

| **Strain and plasmid** | | **Genotype** | **Source** |
| --- | --- | --- | --- |
| **Strains** | | | |
| UMR1 | | ATCC14028-1s Nal^r^ | [1] |
| MAE46 | | UMR1 ∆*ompR101*::Amp^r^ | [2] |
| MAE50 | | UMR1 ΔcsgD101 | [3] |
| MAE108 | | UMR1*fliC5050*::MudJ *fljB5001*::MudCm | [4] |
| **Single mutants** | | | |
| Adra 1f | | UMR1 *adrA101*::MudJ | [5] |
| MAE423 | | UMR1 STM0468::Cm^r^ | [6] |
| MAE425 | | UMR1 STM4264::Cm^r^ | [6] |
| MAE656 | | UMR1 STM3615::Km^r^,Sm^r^ | This study |
| MAE420 | | UMR1 STM3611::Cm^r^ | [6] |
| MAE123 | | UMR1 STM1703::Cm^r^ | [6] |
| MAE279 | | UMR1 STM2410::Cm^r^ | [5] |
| MAE262 | | UMR1 STM2672::Cm^r^ | [5] |
| MAE263 | | UMR1 STM4551::Cm^r^ | [5] |
| MAE272 | | UMR1 STM2123::Cm^r^ | [5] |
| MAE258 | | UMR1 STM1283::Cm^r^ | [5] |
| MAE455 | | UMR1 STM3611::101 | [6] |
| MAE121 | | UMR1 STM3388::Km^r^ | [5] |
| MAE424 | | UMR1 STM1344::Cm^r^ | [6] |
| MAE280 | | UMR1 STM2503::Cm^r^ | [6] |
| MAE422 | | UMR1 STM1827::Cm^r^ | [6] |
| MAE427 | | UMR1 STM1697::Cm^r^ | [6] |
| MAE421 | | UMR1 STM2215::Cm^r^, | [6] |
| MAE454 | | UMR1 ΔSTM4264::101 | [6] |
| MAE259 | | UMR1 STM1987::Cm^r^ | [5] |
| MAE281 | | UMR1 STM3375::Cm^r^ | [5] |
| MAE426 | | UMR1 STM0343::Cm^r^ | [6] |
| **Mutants used for animal experiment** | | | |
| MAE801 | UMR1 STM4264::Cm^r^, Sm^r^ | | This study |
| MAE802 | UMR1 STM1703::Cm^r^, Sm^r^ | | This study |
| MAE803 | UMR1 STM0468::Cm^r^, Sm^r^ | | This study |
| MAE804 | UMR1 STM0385::Cm^r^, Sm^r^ | | This study |
| MAE805 | UMR1 STM2410::Cm^r^, Sm^r^, Amp^r^ | | This study |
| MAE806 | UMR1 STM2672::Cm^r^, Sm^r^, Amp^r^ | | This study |
| MAE807 | UMR1 STM4551::Cm^r^, Sm^r^, Amp^r^ | | This study |
| MAE808 | UMR1 STM2123::Cm^r^, Sm^r^, Amp^r^ | | This study |
| MAE809 | UMR1 STM1283::Cm^r^, Sm^r^ | | This study |
| MAE810 | UMR1 STM3611::Cm^r^, Sm^r^ | | This study |
| MAE811 | UMR1 STM3388::Km^r^, Sm^r^ | | This study |
| MAE812 | UMR1 STM1344::Cm^r^, Sm^r^, Tc^r^ | | This study |
| MAE813 | UMR1 STM2503::Cm^r^, Sm^r^, Tc^r^ | | This study |
| MAE814 | UMR1 STM1827::Cm^r^, Sm^r^, Km ^r^ | | This study |
| MAE815 | UMR1 STM1697::Cm^r^, Sm^r^, Km ^r^ | | This study |
| MAE816 | UMR1 STM2215::Cm^r^, Sm^r^, Tc^r^ | | This study |
| MAE817 | UMR1 STM3611::Cm^r^, Sm^r^, Tc^r^ | | This study |
| MAE818 | UMR1 STM1987::Cm^r^, Sm^r^, Amp^r^ | | This study |
| MAE819 | UMR1 STM3375::Cm^r^, Sm^r^, Tc^r^ | | This study |
| MAE820 | UMR1 STM0343::Cm^r^, Sm^r^, Km ^r^ | | This study |
| MAE827 | UMR1 ATCC 14028-1s Nal^r^, Sm^r^ | | This study |
| SB300 | *S. typhimurium* SL1344 Sm^r^ | | [7] |
| M973 | SL1344 *pagC::pLB02*, Amp^r^ | | [8] |
| SB733 | SL1344 *avrA::aphT*, Km^r^ | | [9] |
| BCB4 | *ttrS* ::mTn10 Tet^r^ | | [10] |
| MAE1759 | UMR1ΔSTM2503*::101* STM3375::Cm | | This study |
| MAE1751 | UMR1 *sipA*::Mini-Tn5-BLAM | | This study |
| MAE1752 | UMR1 *ompR*::Amp *sipA*::Mini-Tn5-BLAM | | This study |
| MAE1755 | UMR1 STM1987::Cm *sipA*::Mini-Tn5-BLAM | | This study |
| MAE1756 | UMR1 ΔSTM4551*::101* *sipA*::Mini-Tn5-BLAM | | This study |
| MAE1757 | UMR1 STM3611::Cm *sipA*::Mini-Tn5-BLAM | | This study |
| MAE1758 | UMR1 ΔSTM4264*::101* *sipA*::Mini-Tn5-BLAM | | This study |
| MAE1760 | UMR1 Δ*csgD101* *sipA*::Mini-Tn5-BLAM | | This study |
| MAE1761 | UMR1Δ*csgD101* STM3611::Cm *sipA*::Mini-Tn5-BLAM | | This study |
| MAE1762 | UMR1 Δ*csgD101* STM4264::Cm *sipA:*:Mini-Tn5-BLAM | | This study |
| **Double mutants** | | | |
| MAE1482 | UMR1 STM0343::101 | | This study |
| MAE1483 | UMR1 STM4551::101 | | This study |
| MAE1484 | UMR1 STM0468::101 | | This study |
| MAE1485 | UMR1 STM3611::101 STM4264::101 | | This study |
| MAE1486 | UMR1 STM3611::101 STM2215:: Cm^r^ | | This study |
| MAE1487 | UMR1 STM4264::101 STM2215:: Cm^r^ | | This study |
| MAE1488 | UMR1 STM0343::101 STM2215:: Cm^r^ | | This study |
| MAE1489 | UMR1 STM4551::101 STM1987:: Cm^r^ | | This study |
| MAE1490 | UMR1 STM4551::101 STM1987::101 | | This study |
| MAE1496 | UMR1 STM0343::101 STM3611:: Cm^r^ | | This study |
| MAE1497 | UMR1 STM0343::101 STM4264:: Cm^r^ | | This study |
| MAE1498 | UMR1 STM0468::Cm STM343::101 | | This study |
| MAE1499 | UMR1 STM2215::cm STM0468::101 | | This study |
| MAE903 | UMR1 STM4264::cm STM3611::101 | | This Study |
| MAE904 | UMR1 STM4264::101 STM0468::Cm^r^ | | This Study |
| MAE432 | UMR1 ΔcsgD101 STM1703::Cm^r^ | | [6] |
| MAE441 | UMR1 ΔSTM4264*::101* STM1283::Cm^r^ | | [6] |
| MAE469 | UMR1 ΔSTM4264*::101* STM3375:: Cm^r^ | | [6] |
| MAE1510 | UMR1 STM4551::101 STM3611::Cm^r^ | | This study |
| MAE1519 | UMR1 STM1987::Cm^r^ STM3611::101 | | This study |
| MAE1521 | UMR1 STM3611::Cm^r^ STM4551::101STM1987::101 | | This study |
| MAE1522 | UMR1 STM4551:Cm^r^ STM4264::101 | | This study |
| MAE1507 | UMR1 STM4264::Cm^r^ STM4551::101STM1987::101 | | This study |
| MAE1523 | UMR1 STM3611::Cm^r^ *bcsA*::101 | | This Study |
| MAE1524 | UMR1 STM3611:: Cm^r^ *csgD*::101 | | [6] |
| MAE1525 | UMR1 STM4264:: Cm^r^ *bcsA*::101 | | This study |
| MAE423 | UMR1 STM4264::cm *csgD*::101 | | [6] |
| MAE1529 | UMR1 STM2503:: Cm^r^ *csgD*::Km^r^ | | This study |
| MAE446 | UMR1 STM1987::Cm^r^ STM4264::101 | | [6] |
| MAE442 | UMR1 ΔSTM4264*::*101 STM1283::Cm^r^ | | [6] |
| MAE469 | UMR1 ΔSTM4264*::*101 STM3375::Cm^r^ | | [6] |
| MAE464 | UMR1 ΔSTM4264:*:*101 STM2503::Cm^r^ | | This study |
| MAE1755 | UMR1 ΔSTM1283*::101*  STM2503 | | This study |
| **Plasmids** | | | |
| pLAFR3 | broad host range vector, IPTG-inducible *lac* promoter; Tc^r^ | | [11] |
| pRGS3 | pLAFR3::STM3611 | | [12] |
| pBAD30 | Arabinose-regulated expression vector, Amp^r^ | | [13] |
| pBAD30*::*4551 | pBAD30 with *Sac*I/*Hin*dIII-ligated STM4551-His6 fragment | | This study |
| pBAD30*::*4551_E267A_ | pBAD30 with STM4551_E267A_ (GGE_267_EF motif changed to GGAEF) | | This study |
| pBAD30*::*1283 | pBAD30 with *Sac*I/*Hin*dIII-ligated STM1283-His6 fragment | | This study |
| pBAD30*::*1283_D425A_ | pBAD30 with STM1283 _D425A_ (GGD_425_EF motif changed to GGAEF) | | This study |
| pRGS1 | pBAD30 with STM3611 | | [12] |
| pBAD30::3611_K179A_ | pBAD30 with STM3611_K179A_ | | This study |
| pUMR15 | pBAD30::*csgD* | | [3] |

**References**

1. Römling U, Bian Z, Hammar M, Sierralta WD, Normark S (1998) Curli fibers are highly conserved between *Salmonella typhimurium* and *Escherichia coli* with respect to operon structure and regulation. J Bacteriol 180: 722-731.

2. Römling U, Sierralta WD, Eriksson K, Normark S (1998) Multicellular and aggregative behaviour of *Salmonella typhimurium* strains is controlled by mutations in the *agfD* promoter. Mol Microbiol 28: 249-264.

3. Römling U, Rohde M, Olsen A, Normark S, Reinköster J (2000) AgfD, the checkpoint of multicellular and aggregative behaviour in *Salmonella typhimurium* regulates at least two independent pathways. Mol Microbiol 36: 10-23.

4. Rochon M, Romling U (2006) Flagellin in combination with curli fimbriae elicits an immune response in the gastrointestinal epithelial cell line HT-29. Microbes Infect 8: 2027-2033.

5. Kader A, Simm, R., Gerstel, U., Morr, M., Römling, U. (2006) Hierarchical involvement of various GGDEF domain proteins in rdar morphotype development of *Salmonella enterica* serovar Typhimurium. Mol Microbiol 60: 602-616.

6. Simm R, Lusch A, Kader A, Andersson M, Römling U (2007) Role of EAL-containing proteins in multicellular behavior of *Salmonella enterica* serovar Typhimurium. J Bacteriol 189: 3613-3623.

7. Hoiseth SK, Stocker BA (1981) Aromatic-dependent *Salmonella typhimurium* are non-virulent and effective as live vaccines. Nature 291: 238-239.

8. Stecher B, Hapfelmeier S, Muller C, Kremer M, Stallmach T, et al. (2004) Flagella and chemotaxis are required for efficient induction of *Salmonella enterica* serovar Typhimurium colitis in streptomycin-pretreated mice. Infect Immun 72: 4138-4150.

9. Hardt WD, Galan JE (1997) A secreted *Salmonella* protein with homology to an avirulence determinant of plant pathogenic bacteria. Proc Natl Acad Sci U S A 94: 9887-9892.

10. Hensel M, Hinsley AP, Nikolaus T, Sawers G, Berks BC (1999) The genetic basis of tetrathionate respiration in *Salmonella typhimurium*. Mol Microbiol 32: 275-287.

11. Staskawicz B, Dahlbeck D, Keen N, Napoli C (1987) Molecular characterization of cloned avirulence genes from race 0 and race 1 of *Pseudomonas syringae* pv. glycinea. J Bacteriol 169: 5789-5794.

12. Simm R, Morr, M., Kader, A., Nimtz, M., Römling, U. (2004) GGDEF and EAL domains inversely regulate cyclic di-GMP levels and transition from sessility to motility. Mol Microbiol 53: 1123-1134.

13. Guzman LM, Belin D, Carson MJ, Beckwith J (1995) Tight regulation, modulation, and high-level expression by vectors containing the arabinose PBAD promoter. J Bacteriol 177: 4121-4130.
