# Supplementary material for: Complex c-di-GMP Signaling Networks Mediate Transition between Virulence Properties and Biofilm Formation in Salmonella enterica Serovar Typhimurium
Source: PLoS One. 2011 Dec 2;6(12):e28351. doi: 10.1371/journal.pone.0028351 (PMC3229569; doi:10.1371/journal.pone.0028351)
Supplement: Table S2 — Primers used in this study. (DOCX) [file pone.0028351.s009.docx]

**Table S2.** Primers used in this study

| Primer | Sequence |
| --- | --- |
| Primers for control of mutants | |
| STM0343-KO-check-forward | CTGCGCAGAACGGGTATGA |
| STM0343_Contr_Rev | CAATCAGATAATTGTTCTGACT |
| STM0468-KO-check-forward | AGGATGAATATCAGGTTTCAT |
| STM0468_Contr_Rev | AAGCAGCCAAGGCAGATGA |
| STM1344-KO-check-forward | TCTGGCAGGCACGGCGAA |
| STM1344_Contr_Rev | GGGCAGTAAAAGACAGGGT |
| STM3611-KO-check-forward | CAATCACGGCAATCTTTTTAG |
| STM3611_Contr_Rev | TGTGATGGCGGCGGTTATT |
| STM2215-KO-check-forward | GTCGAGTTCTGAGCCGCA |
| STM2215_Contr_Rev | GAAGCATTACGCGCGCAAT |
| STM1827-KO-check-forward | TTGGATAAAGTCATCGAAACGAT |
| STM1827_Contr_Rev | TGCCCACGACGAAGAGGAA |
| STM4264-KO-check-forward | GACACCAGTGGTTATGGCA |
| STM4264_Contr_Rev | ATCGCACTCCCAGCGATTA |
| STM1697-KO-check-forward | GACTCTTGTGGCTAAGGTAT |
| STM1697_Contr_Rev | GCATGGCGAAAGGATCGC |
| STM2123-KO-check-forward | CACTGATGAGACTGATCAGT |
| STM2410-KO-check-forward | GTTCGATCCCGCTTAGCTC |
| STM1987-KO-check-Forward | CGGTTGTCGTTAAAGGGCTA |
| STM1987_KO_Contr_Rev | AAGGCGCGCGTGCGGACG |
| STM1283-KO-check-Forward | TTCTCCTGGGTTAATAAAGCC |
| STM1283_KO_Contr_Rev | ATTCTCATCGCTATGGTAAACT |
| STM3375-KO-check-Forward | GTGCAGTTCGTTTCCTTTGTT |
| STM3375_KO_Contr_Rev | GTCCTGCTGTTCTAAGATCAA |
| STM2503-KO-check-Forward | GTAATAATTGACTTAAATCAA |
| STM2503_KO_Contr_Rev | CTAATATTGAGGAAGAAAG |
| STM4551-KO-check-Forward | GCGGTCTATTATAGCAGTCG |
| STM4551_KO_Contr_Rev | CGCCAGATAATATCGTCAGC |
| csgD_control | TGTTTAACACGCATGACAGC |
| CsgD_KO_Contr_Rev | CGTAATTGGACCTGTCGTGTT |
| STMSipA458-Forward | ACGGCAGGGCTGTGACCAA |
| SeqstopA-reverse | TCAAGGATCTTACCGCTGTT |
|  |  |
| Primer for gene cloning | |
| 4551-fw-SacI | 5’‑ttaaagagctccaccgataaataacgacaattacccatgacaaccccatcctggcggtc-3’ |
| 4551-rev-6his-HindIII | 5’‑aaaaaagctttcagtgatggtgatggtgatgtagggcgcgcatgtcgtcgctgaccagaatg-3’ |
| 1283-fw-SacI | 5’‑aaaagagctccgactttcagggctgtagttcatcatgaatttgcatcataaag-3’ |
| 1283-rev-6his-HindIII | 5’‑ttttaagcttctagtgatggtgatggtgatgtgatgaacgatgttgtttttgttgtttgttc‑3’ |
|  |  |
| Primer for generation of mutation in the GGDEF motif | |
| 4551-GGAEF-fw | 5’‑gtcgtctgccgttttggcggggcggaatttc‑3’ |
| 4551-GGAEF-rev | 5’‑gtggtcagcaacaccagaaattccgccccgcc‑3’ |
| new-GGAEF-1283 -fw | 5’‑gccattcgcctcggcggcgcggagttctgtattattc-3’ |
| new-GGAEF-1283-rev | 5’‑gaataatacagaactccgcgccgccgaggcgaatggc‑3’ |
|  |  |
| Primer for generation of mutation in the KIDRTF submotif | |
| 3611_K179A forw | 5’-actacatt*gcg*gtggcgcg-3’ |
| 3611_K179A Rev | 5’-cgcgccac*cgc*aatgtagt-3’ |

Underlined sequences indicated recognition sites for restriction enzymes; sequences in italics indicate exchanged nucleotides.
